# Detecting Blood-Based Biomarkers in Metastatic Breast Cancer: A Systematic Review of Their Current Status and Clinical Utility

**DOI:** 10.3390/ijms18020363

**Published:** 2017-02-09

**Authors:** A. M. Sofie Berghuis, Hendrik Koffijberg, Jai Prakash, Leon W. M. M. Terstappen, Maarten J. IJzerman

**Affiliations:** 1 Health Technology and Services Research, University of Twente, Enschede 7500 AE, The Netherlands; h.koffijberg@utwente.nl (H.K.); m.j.ijzerman@utwente.nl (M.J.I.); 2 Biomaterials Science and Technology, University of Twente, Enschede 7500 AE, The Netherlands; j.prakash@utwente.nl; 3 Medical Cell Biophysics, University of Twente, Enschede 7500 AE, The Netherlands; l.w.m.m.terstappen@utwente.nl

**Keywords:** metastatic breast cancer, liquid biopsy, circulating biomarkers, blood-based biomarkers, circulating tumor cells (CTCs), utility, developmental stages, development evaluation and application chart (DEAC)

## Abstract

Reviews on circulating biomarkers in breast cancer usually focus on one single biomarker or a selective group of biomarkers. An overview summarizing the discovery and evaluation of all blood-based biomarkers in metastatic breast cancer is lacking. This systematic review aims to identify the available evidence of known blood-based biomarkers in metastatic breast cancer, regarding their clinical utility and state-of-the-art position in the validation process. The initial search yielded 1078 original studies, of which 420 were assessed for eligibility. A total of 320 studies were included in the final synthesis. A Development, Evaluation and Application Chart (DEAC) of all biomarkers was developed. Most studies focus on identifying new biomarkers and search for relations between these biomarkers and traditional molecular characteristics. Biomarkers are usually investigated in only one study (68.8%). Only 9.8% of all biomarkers was investigated in more than five studies. Circulating tumor cells, gene expression within tumor cells and the concentration of secreted proteins are the most frequently investigated biomarkers in liquid biopsies. However, there is a lack of studies focusing on identifying the clinical utility of these biomarkers, by which the additional value still seems to be limited according to the investigated evidence.

## 1. Introduction

### 1.1. Breast Cancer Survival

Globally, breast cancer is the most commonly diagnosed form of cancer among women. Clinical management has improved over the last years, and the development of genetic tests such as Mammaprint and OncoTypeDX have proven to guide treatment in early stage breast cancer. Although the current 5-year survival for primary breast cancer is relatively high (ranging from 80% to 92% in different populations) [1], survival rates decrease to less than 25% when the disease becomes metastatic [1,2]. The most important factor to increase survival for those suffering from metastatic breast cancer, is to prescribe a treatment that has the most likelihood of being effective, guided by the tumor cell characteristics [3,4]. To select the most effective treatment once the metastatic lesions have been detected, it is essential to obtain accurate information on the characteristics of the tumor cells at the time therapy is to be initiated [5].

### 1.2. Detection and Treatment of Metastatic Lesions

Technical advances in the molecular characterization of cells has already lead to accurate predictions of survival and treatment efficacy. However, these molecular characterizations require high-quality biopsies, which cannot always be obtained from the primary tumor [6]. Alternatively, taking a biopsy of the metastatic lesion is either difficult or even impossible, for example, due to its location, or the inability to visualize that location with the currently used imaging techniques [6,7,8]. Furthermore, previous research has shown that molecular aberrations of the primary tumor may differ from that of the metastatic lesion and different metastatic lesions can have different characteristics [9]. Therefore, there remains a need for new tests which are sufficiently sensitive and reflect the composition of the tumor at all sites to guide treatment of metastatic disease.

### 1.3. The Use of Blood-Based Biomarkers

A possible way of enabling better treatment response monitoring or treatment guidance is the use of blood-based biomarkers or liquid biopsies [10]. A large number of single blood-based biomarkers can be distinguished in the blood, of which the most commonly known soluble proteins are Human Epidermal Growth Factor Receptor 2 (HER2), Cancer Antigen 15-3 (CA 15-3), Carcinoembryonic Antigen (CEA) and MUC1 [11]. Furthermore, all kinds of gene expression patterns or mutations can be extracted from circulating mRNA or circulating free DNA [8,12]. However, not only proteins or gene expression patterns yield prognostic or predictive information, even complete cells found in the blood—such as Circulating Tumor Cells (CTCs) or Cancer Associated Fibroblasts (CAF)—provide this type of information.

Although a range of different biomarkers is known, it is far more difficult to evaluate their usefulness for treatment targeting or prognosis of disease. It therefore is required to develop a classification, both to determine biomarkers with clinical utility and to prioritize future research. For clinical decision making, there are different ways of classifying diagnostic information [10]. Classifications focus, for example, on prognostic or predictive ability, or on a classification according to specific hallmarks of cancer [12].

### 1.4. Evidence on the Utility of Biomarkers

Up to now, the literature is not clear about the clinical utility of biomarkers in breast cancer. Several systematic reviews on blood-based biomarkers have been published yet [10,13]. However, these studies usually focus on one single biomarker or a selective group of biomarkers. These reviews are helpful to understand specific molecular pathways of oncogenesis, on specific prognostic information and all other outcomes they are related to, or on both.

An overview summarizing the discovery and evaluation of blood-based biomarkers for metastatic disease, in terms of their current status and future potential for clinical application, is still lacking. Therefore, this systematic review focusses on identifying known biomarkers, the available evidence regarding their clinical utility and exploring the current state-of-the-art in the validation process of all blood-based biomarkers in metastatic breast cancer. The review aims to identify a set of blood-based biomarkers that may have substantial future potential. Whereas it is common to focus on outcomes in terms of effectiveness, this review instead focusses on the developmental stage as the primary outcome measure of the included studies. First, all blood-based biomarkers will be identified and classified according to their developmental stage (e.g., from discovery to clinical utility). Second, the set of biomarkers with the highest future potential for clinical application will be identified by the number of studies that have been performed in each of the developmental stages.

### 1.5. Conclusions

The main aim of research on blood-based biomarkers in metastatic breast cancer is the identification of new biomarkers or relations of these biomarkers with other original molecular tumor characteristics. Especially gene expression within CTCs is investigated frequently. However, there still is a lack of studies identifying the clinical utility of these biomarkers. Thereby, the additional value for these biomarkers seems to be still limited according to the investigated evidence.

## 2. Results

### 2.1. Search Results

**Figure 1:**
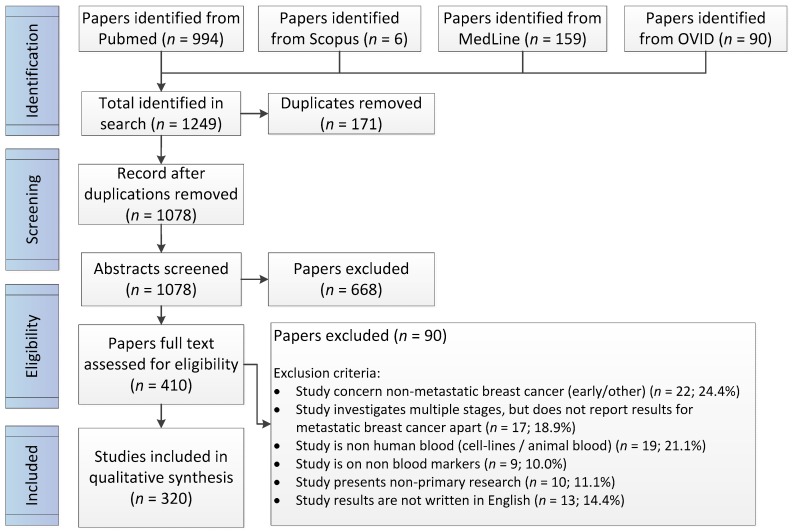
PRISMA Flow Diagram.

The initial search resulted in a total of 1249 studies from all databases searched. After screening all abstracts, 410 studies were further assessed for eligibility. During the assessment for eligibility, 91 studies were excluded. A total of 320 studies were included in this review. The full list of all studies that were included is presented in Appendix C. Most studies were excluded because the biomarkers investigated were not extracted from metastatic breast cancer patients (*n* = 22; 24.4%), because the blood used in the detection of the biomarker was non-human or was injected with a cell line that had just metastatic potential (*n* = 19; 21.1%) or because the study investigated multiple stages of breast cancer but had not reported conclusions for metastatic breast cancer separately (*n* = 17; 18.9%). The flow diagram of the search is presented in Figure 1.

### 2.2. Study Characteristics

**Figure 2:**
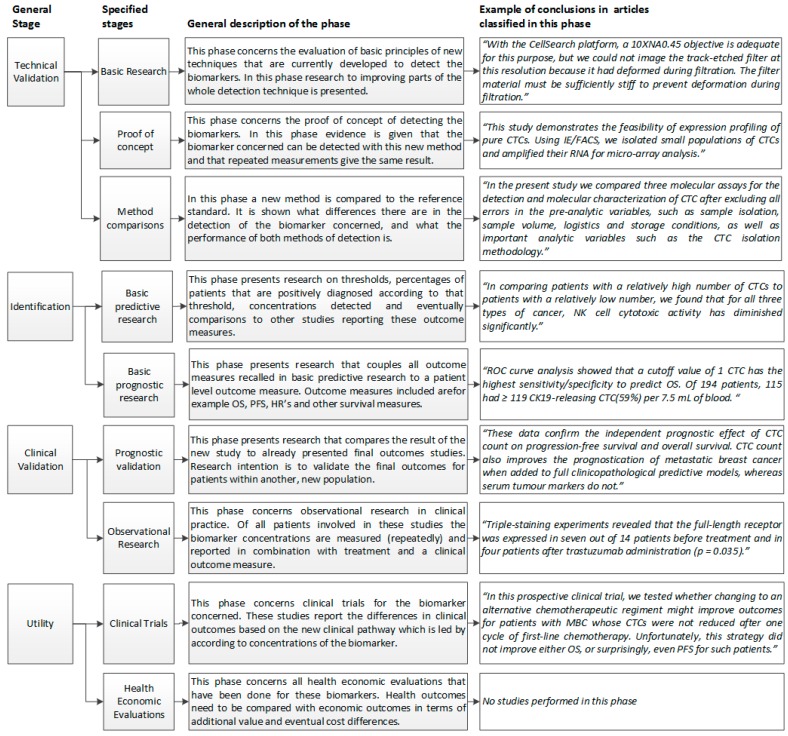
Stages of clinical translation in biomarker discovery [14,15,16,17,18,19,20,21].

For each study, the data were extracted and two classifications were made. First, the biomarkers were classified in one of the four general categories. Second, studies were classified in one of the pre-defined developmental stage categories, as defined in Figure 2. To illustrate the classification more clearly, citations of those studies which were classified as being in one of these phases are given in the right column of Figure 2.

### 2.3. Results According to Developmental Phase

**Figure 3:**
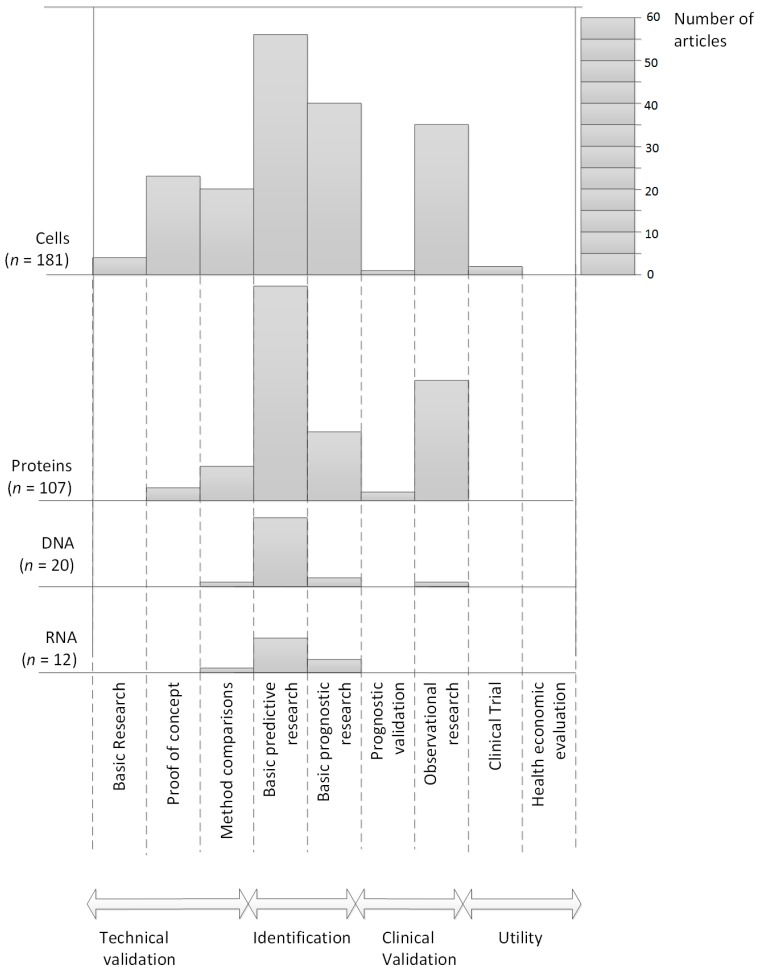
Development, Evaluation and Application Chart.

Figure 3 presents the DEAC with the distribution of studies over developmental phases. From this figure it is apparent that most studies focused on the identification phase. This means that most studies focus on finding relationships between the concentration of the biomarker, in relation to a new or existing threshold and furthermore, try to evaluate this against an outcome measure in terms of survival (e.g., Overall Survival (OS), Progression Free Survival (PFS) or survival in months). This phase is split up over two sub phases, namely basic predictive and basic prognostic research. For predictive research, only the concentrations in a subgroup of metastatic breast cancer patients were reported. For prognostic research, these concentrations were linked to an outcome measure related to survival (OS or PFS).

### 2.4. Results per Biomarker

The general biomarker category in which most studies were performed on blood-based biomarkers in metastatic breast cancer, concerned whole cells in the blood (*n* = 181; 56.6%). CTCs made up a large part of this. In 85.1% (*n* = 154) of all included studies CTC enumeration was performed. In 42.5% of all included studies (*n* = 136), also genetic profiling for these cells had been done. The markers most frequently investigated are presented in Table 1.

Only those biomarkers for which 5 or more studies have been performed are included in the table. This cut-off had been chosen because these markers represent the most frequently investigated biomarkers. The frequency by which biomarkers are investigated is presented in Table 2, which presents that only 9.8% of all biomarkers is investigated in more than 5 studies. A detailed overview presenting the amount of studies performed for each single biomarker, including an overview of the amount of studies in each developmental stage is presented in Appendix D.

The percentages shown in Table 1 present the percentage of total studies that investigated that single marker. The second general biomarker category on which a relatively large amount of studies have been performed (*n* = 107; 33.4%) are proteins. Within this category most research has been focusing on 4 proteins, which are: CA15-3 (*n* = 22; 20.5%), soluble HER2 (*n* = 19; 17.8%), Vascular Endothelial Growth Factor (VEGF) (*n* = 18; 16.8%) and Vascular Endothelial Growth Factor Receptor (VEGFR) (*n* = 14; 13.1%). As discussed before, frequently studies are focusing on investigating multiple biomarkers instead of single biomarkers. As presented in Table 1, a total of 51 studies have investigated CA15-3. This means that also research which mainly focuses on one of the other biomarker categories investigates CA15-3. The same differences in the amount of studies performed were seen for HER2 and VEGF.

### 2.5. Results on the Number of Studies Performed

Summarized over all general biomarker categories, the total amount of studies included in the results synthesis is 320 as presented in Figure 2. In these studies a total of 275 single biomarkers have been investigated. The average number of studies performed on one single biomarker is 2.6 (range 1–154 studies). In Table 2 results are presented for frequency by which the study investigated a number of biomarkers. Table 2 shows that for 68.8% of all the biomarkers only one study has investigated that particular biomarker. For 13.8% of all biomarkers two studies have investigated that biomarker.

## 3. Discussion

In this paper we present a broad overview of research on blood-based biomarkers in metastatic breast cancer, performed since 2006. Of the included studies, most focused on detecting whole cells in the blood, with a focus on the enumeration or genetic characterization of circulating tumor cells. Considering the classification into developmental stages, the identification stage is the stage during which most research has been performed. Most studies focus on the identification phase, in which they investigate the ability to detect particular biomarkers in the blood and are trying to find connections between these concentrations and potential outcome measures in terms of survival. For proteins CA15-3, soluble HER2, VEGF and VEGFR have been investigated most frequently. However, for CTCs there have been clinical trials, but not for one of these proteins since 2006.

In terms of developmental stages, we expected that the amount of research performed would follow some kind of trend over time. It was expected that per biomarker there would be a substantial amount of studies focusing on the early developmental stages (technical validation), with decreasing numbers of studies the further the research for that particular biomarker proceeded in the developmental process. However, the DEAC shows that this trend does not exist for blood-based biomarkers in metastatic breast cancer. The DEAC shows that the number of studies performed increase until they reach the identification phase, and decrease afterwards. Therefore, it seems that the technical validation and clinical validation phase are currently less performed than research in the identification phase. Another observation from the DEAC is the low amount of research performed in the prognostic validation phase, suggesting this phase is not receiving sufficient attention. However, this may well be due to the fact that the initial search was limited to articles published since 2006, so that a limited amount of studies concerning some of the developmental phases were found. It might have been that specific phases which seemed to have had insufficient attention for several biomarkers were investigated before 2006. In addition, some information might have been missed, as publication bias may have occurred due to excluding non-English studies.

Furthermore, biomarker research may have been performed in a commercial setting or for stakeholders intent to guide internal research and development decisions. As such, selective reporting may occur by which not all findings might have been published. The same holds for studies with negative findings on (some subset) of investigated biomarkers, as it is known that such results are harder to publish than positive findings. As we did not investigate a single outcome measure, no standard methods are available to assess the ensuing risk of bias in our results. Even though the intention of reports might be to inform about recent developments, other stakeholders might use this information differently. Therefore, it seems valuable for future research to be able to have access to all information that was, or can possibly be extracted from the blood samples. Future research should pay attention to selective reporting before publishing, or ensure that samples are publicly available via biobanks.

## 4. Materials and Methods

This systematic review of blood-based biomarkers in metastatic breast cancer was performed according to the PRISMA guidelines [22]. A review protocol was used and is presented in Appendix A. This review was not registered in the PROSPERO database. All types of studies were included in the initial review, as the aim of this review is to identify the best available evidence exploring the position in the development process of all blood-based biomarkers in metastatic breast cancer. Since all types of primary research studies were included, it was not required that the intervention, control or specific outcome measure was reported in the initial search. Therefore, no specific study characteristics or PICO-statement for inclusion criteria was used. The only restriction applied to the search concerned a time constraint, as studies published since 1 January 2006 were included. Databases that were searched are PubMed, Scopus and OVID. Additionally, articles found by cross-referencing or hand search were included in the initial search. The initial search was performed in June 2016 and updated on 1 December 2016. The detailed search terms applied are presented in Appendix B.

After the initial search and removal of duplicate papers, abstracts were scanned for relevance. Abstracts of articles that either did not present non-primary research data or concerned topics not of interest here (such as, other cancer types, only other stages of breast cancer, and non-blood-based biomarkers—e.g., biomarkers that can be found in other body fluids) were excluded from the full-text review. All abstracts were processed by one reviewer (A. M. Sofie Berghuis) and were discussedwith a second reviewer (Hendrik Koffijberg) if necessary.

Full texts of all included papers were assessed for eligibility by one reviewer (A. M. Sofie Berghuis). All studies were then categorized according to the 10 pre-defined developmental stage and per general biomarker type. Four general developmental stages were identified, namely technical validation, identification, clinical validation and clinical utility. A full description of all pre-defined developmental stages is presented in Figure 1. Data was then classified in four general types of biomarkers, namely cells, proteins, circulating DNA and circulating RNA. Final classification of studies was discussed with a second reviewer (Hendrik Koffijberg) if classification in either one of the categories was unclear to the first reviewer (A. M. Sofie Berghuis). For studies on which there was no consensus between these two reviewers, a third reviewer reclassified the study (Maarten J. IJzerman).

### 4.1. Article Processing

Quantitative and qualitative data was manually extracted from the included studies and structured in Excel (version 2013) in pre-defined and labeled columns. The following information was extracted from all the included studies:

- General biomarker classification (classification in one of the four categories: cells, proteins, circulating DNA or circulating RNA)
- Developmental stage (classification according to the stages and general descriptions of these stages shown in Figure 1)
- Specific biomarker name
- Type of test used to quantify or detect biomarker (e.g., ELISA, CellSearch, etc.)
- Whether—and if so, which—survival data was presented (Overall Survival, Progression Free Survival, survival in months)

Given the focus on the translation of biomarkers to clinical practice, the results of all included studies were summarized according to the number of studies performed per developmental stage for each general biomarker category. Results for all single biomarkers were summarized per general biomarker category as studies might investigate more than one biomarker. For all single biomarkers it was determined how many studies investigated that biomarker and in which stage of translation the biomarker was identified. For each general biomarker category it was investigated how many studies presented results on the full range of single biomarkers found.

### 4.2. Synthesis of Results

Results were presented in a Development, Evaluation and Application Chart (DEAC) that was developed specifically for this review. This figure gives a broad overview of the development of biomarkers in each of the predefined stages of clinical translation. Specifically, the figure shows four bar diagrams above each other, one diagram for each of the general biomarker categories. Each vertical bar, per diagram, represents a developmental stage. The bars are displayed to represent the different stages in the translation, starting with the most basic (developmental) research on the left side and more advanced (evaluation) research (such as clinical trials or health economic evaluations) presented on the right side. The height of the bars reflects the number of included studies. This figure therefore gives an overview of the number of studies published on each of the general biomarker categories according to the developmental stage timeline.

## 5. Conclusions

Since 2006, a substantial amount of research has been done to investigate the potential role of blood-based biomarkers in metastatic breast cancer. There seems to be a focus on research toward the use of CTCs, as most studies investigate these, whether in combination with other markers or as a single marker. The current emphasis of investigating these biomarkers seems to be on developing new techniques or finding new biomarkers that might have predictive or prognostic value, as most studies focus on the identification phase. There is a lack of studies focusing on clinical utility of these biomarkers. This might be because these studies have not yet been performed or suffer from publication bias. However, the lack of studies investigating the utility of blood-based biomarkers causes the additional value in terms of clinical utility, health outcomes or health care efficiency to still be limited according to the investigated evidence.

## Figures and Tables

**Table 1:** Most frequently investigated biomarkers of all studies included.

Biomarker *	Number of Articles	% of Included Studies	End Stage	Number of Studies at End Stage
ALDH1	5	1.6%	Observational	1
CA15-3	51	15.9%	Observational	6
CEA	19	5.9%	Observational	1
CK19	6	1.9%	Observational	1
CTC enumeration	154	48.1%	Clinical trial	29
EGFR	15	4.7%	Observational	6
ER	13	4.1%	Basic prognostic	3
HER2	61	19.1%	Observational	15
PIK3CA	13	4.1%	Observational	1
PR	7	2.2%	Basic prognostic	2
RASSF1A	6	1. 9%	Basic predictive	5
THBS-1	9	2.8%	Observational	5
TP53	5	1.6%	Basic prognostic	1
TWIST	7	2.2%	Observational	2
VEGF	22	6.9%	Observational	15
VEGFR	13	4.1%	Observational	12
Vimentin	6	1.9%	Basic prognostic	1

* The abbreviations used are standard abbreviations. Corresponding gene identities encoding for these biomarkers are presented in Appendix D.

**Table 2:** Number of studies in which single biomarkers are investigated.

Number of Studies that Investigated a Specific Biomarker	Frequency	% of All Included Studies
1	190	68.8%
2	38	13.8%
3	12	4.3%
4	9	3.3%
5–10	19	6.9%
>10	8	2.9%

**Figure A1:**
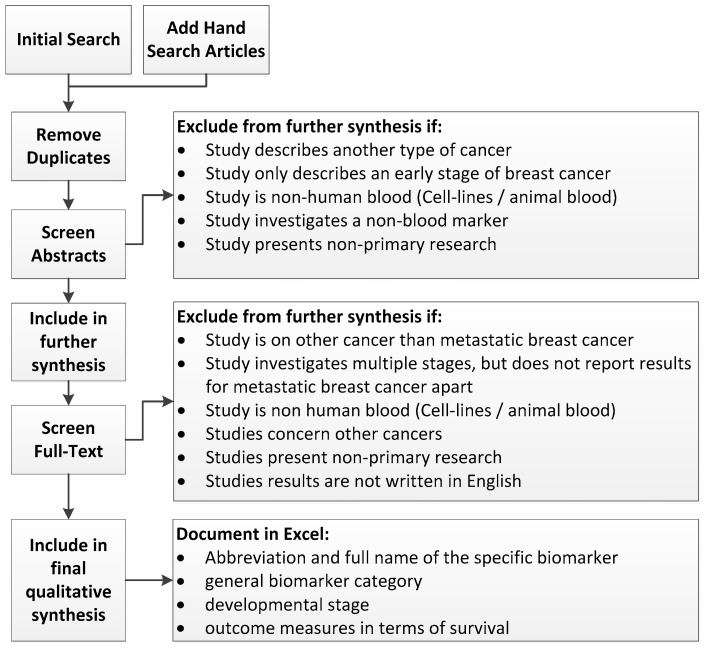
Review Protocol.

## Abbreviations

CA15-3	Cancer Antigen 15-3
CTC	Circulating Tumor Cell
DEAC	Development, Evaluation and Application Chart
PFS	Progression Free Survival
HER2	Human Epidermal Growth Factor Receptor 2
OS	Overall Survival
VEGF	Vascular Endothelial Growth Factor
VEGFR	Vascular Endothelial Growth Factor Receptor

## Appendix A. Review Protocol

The steps described in Figure A1 were used as a review protocol.

In the abstract, screening the abstracts were only excluded when they explicitly presented that they contained information on one of the five exclusion criteria. If it was doubted whether results for blood-based biomarkers could have been presented in the text due to vague descriptions, the article was included for full text review. An example of this is the use of “Advanced breast cancer” as study population. For advanced often stage IIIB/IIIC and stage IV is meant. However, several abstracts did not present which stages they exactly investigated. These abstracts therefore were included.

Non-primary research that was excluded from further synthesis was saved in a separate folder, so these can be easily accessed after the complete search. Thereby, comparisons between the results found and the results that were already presented could easily be made.

## Appendix B. Search Terms

Below the full electronic search strategy is presented, i updated until 1 December 2016.

### Appendix B.1. Final Search Term PubMed (n = 994)

(((Metastatic[tiab] OR Advanced [tiab] OR Late stage[tiab] OR Malign*[tiab] OR Stage IV[tiab] OR Secondary[tiab]) AND (Breast cancer[majr] OR Breast neoplasm[majr] OR Breast carcinoma[majr] OR Mamma carcinoma[majr] OR Breast tumor[majr] OR Breast tumour[majr] OR “breast neoplasms”[Mesh])) AND ((Blood based[tw] OR Circulating[tw] OR Plasma[tw] OR Liquid[tw] OR Serum[tw] OR Serum based[tw] OR Extracellular[tw]) AND (blood[tw] AND (Marker*[tw] OR Biomarker*[tw] OR Biops*[tw] OR Tumor Cell*[tw] OR Tumor micro particle*[tw] OR Tumor particle*[tw] OR Tumor vesicle*[tw] OR Tumour Cell*[tw] OR Tumour micro particle*[tw] OR Tumour particle*[tw] OR Tumour vesicle*[tw] OR Tumor marker*[tw] OR Tumour marker*[tw] OR Tumor cell cluster*[tw] OR Tumour cell cluster*))))

### Appendix B.2. Final Search Term Scopus (n = 6)

(((ALL(blood based OR circulating OR plasma OR liquid OR serum OR serum based OR extracellular)) AND (ALL (marker OR biomarker OR biops OR tumor cell OR tumor micro particle OR tumor particle OR tumor vesicle OR tumour cell OR tumour micro particle OR tumour particle OR tumour vesicle OR tumor marker OR tumour marker OR tumor cell cluster OR tumour cell cluster))) AND (ALL (blood))) AND ((ABS (metastatic OR advanced OR late stage OR malign OR stage iv OR secondary)) AND (ALL (breast cancer OR breast neoplasm OR breast carcinoma OR mamma carcinoma OR breast tumor OR breast tumour)))

### Appendix B.3. Final Search Terms OVID (n = 90)

(((ALL (blood based OR circulating OR plasma OR liquid OR serum OR serum based OR extracellular)) AND (ALL (marker OR biomarker OR biops OR tumor cell OR tumor micro particle OR tumor particle OR tumor vesicle OR tumour cell OR tumour micro particle OR tumour particle OR tumour vesicle OR tumor marker OR tumour marker OR tumor cell cluster OR tumour cell cluster))) AND (ALL (blood))) AND ((ABS (metastatic OR advanced OR late stage OR malign OR stage iv OR secondary)) AND (ALL (breast cancer OR breast neoplasm OR breast carcinoma OR mamma carcinoma OR breast tumor OR breast tumour)))

### Appendix B.4. Final Search Terms Medline (n = 159)

(((TI = (metastatic OR advanced OR late stage OR malign OR stage iv OR secondary)) AND (TI = (breast cancer OR breast neoplasm OR breast carcinoma OR mamma carcinoma OR breast tumor OR breast tumour))) AND ((TI = (blood based OR circulating OR plasma OR liquid OR serum OR serum based OR extracellular)) AND (TI = (marker OR biomarker OR biops OR tumor cell OR tumor micro particle OR tumor particle OR tumor vesicle OR tumour cell OR tumour micro particle OR tumour particle OR tumour vesicle OR tumor marker OR tumour marker OR tumor cell cluster OR tumour cell cluster)))) AND LANGUAGE: (English) AND DOCUMENT TYPES: (Article)

Indexes = SCI-EXPANDED, SSCI, A&HCI, ESCI Timespan = 2006–2016

## Appendix C. Overview of All Included Studies

1. Zhang SH, Li L, Wang T, Bian L, Hu HX, Xu CH, et al. Real-time HER2 status detected on circulating tumor cells predicts different outcomes of anti-HER2 therapy in histologically HER2-positive metastatic breast cancer patients. BMC cancer. 2016;16.
2. Windrichova J, Fuchsova R, Kucera R, Topolcan O, Fiala O, Finek J, et al. Testing of a Novel Cancer Metastatic Multiplex Panel for the Detection of Bone-metastatic Disease—A Pilot Study. Anticancer research. 2016;36(4):1973-8.
3. Shiomi-Mouri Y, Kousaka J, Ando T, Tetsuka R, Nakano S, Yoshida M, et al. Clinical significance of circulating tumor cells (CTCs) with respect to optimal cut-off value and tumor markers in advanced/metastatic breast cancer. Breast cancer (Tokyo, Japan). 2016;23(1):120-7.
4. Reijm EA, Sieuwerts AM, Smid M, Bolt-de Vries J, Mostert B, Onstenk W, et al. An 8-gene mRNA expression profile in circulating tumor cells predicts response to aromatase inhibitors in metastatic breast cancer patients. BMC cancer. 2016;16.
5. Perroud HA, Alasino CM, Rico MJ, Mainetti LE, Queralt F, Pezzotto SM, et al. Metastatic breast cancer patients treated with low-dose metronomic chemotherapy with cyclophosphamide and celecoxib: clinical outcomes and biomarkers of response. Cancer chemotherapy and pharmacology. 2016;77(2):365-74.
6. Peng CK, Chen HD, Wallwiener M, Modugno C, Cuk K, Madhavan D, et al. Plasma S100P level as a novel prognostic marker of metastatic breast cancer. Breast cancer research and treatment. 2016;157(2):329-38.
7. Peng C, Wallwiener M, Rudolph A, Cuk K, Eilber U, Celik M, et al. Plasma hyaluronic acid level as a prognostic and monitoring marker of metastatic breast cancer. International journal of cancer. 2016;138(10):2499-509.
8. Nakauchi C, Kagara N, Shimazu K, Shimomura A, Naoi Y, Shimoda M, et al. Detection of TP53/PIK3CA Mutations in Cell-Free Plasma DNA From Metastatic Breast Cancer Patients Using Next Generation Sequencing. Clinical breast cancer. 2016;16(5):418-23.
9. Mu ZM, Benali-Furet N, Uzan G, Znaty A, Ye Z, Paolillo C, et al. Detection and Characterization of Circulating Tumor Associated Cells in Metastatic Breast Cancer. International journal of molecular sciences. 2016;17(10).
10. Matthew EM, Zhou L, Yang Z, Dicker DT, Holder SL, Lim B, et al. A multiplexed marker-based algorithm for diagnosis of carcinoma of unknown primary using circulating tumor cells. Oncotarget. 2016;7(4):3662-76.
11. Lee CK, Davies L, Gebski VJ, Lord SJ, Di Leo A, Johnston S, et al. Serum Human Epidermal Growth Factor 2 Extracellular Domain as a Predictive Biomarker for Lapatinib Treatment Efficacy in Patients With Advanced Breast Cancer. Journal of clinical oncology: official journal of the American Society of Clinical Oncology. 2016;34(9):936-44.
12. Kristiansen S, Nielsen D, Soletormos G. Detection and monitoring of hypermethylated RASSF1A in serum from patients with metastatic breast cancer. Clinical epigenetics. 2016;8:35.
13. Hyun KA, Goo KB, Han H, Sohn J, Choi W, Kim SI, et al. Epithelial-to-mesenchymal transition leads to loss of EpCAM and different physical properties in circulating tumor cells from metastatic breast cancer. Oncotarget. 2016;7(17):24677-87.
14. Hainsworth JD, Murphy PB, Alemar JR, Daniel BR, Young RR, Yardley DA. Use of a multiplexed immunoassay (PRO Onc assay) to detect HER2 abnormalities in circulating tumor cells of women with HER2-negative metastatic breast cancer: Lack of response to HER2-targeted therapy. Breast cancer research and treatment. 2016;160(1):41-9.
15. Gogoi P, Sepehri S, Zhou Y, Gorin MA, Paolillo C, Capoluongo E, et al. Development of an Automated and Sensitive Microfluidic Device for Capturing and Characterizing Circulating Tumor Cells (CTCs) from Clinical Blood Samples. PloS one. 2016;11(1):e0147400.
16. Gasch C, Oldopp T, Mauermann O, Gorges TM, Andreas A, Coith C, et al. Frequent detection of PIK3CA mutations in single circulating tumor cells of patients suffering from HER2-negative metastatic breast cancer. Molecular oncology. 2016;10(8):1330-43.
17. Deutsch TM, Riethdorf S, Nees J, Hartkopf AD, Schonfisch B, Domschke C, et al. Impact of apoptotic circulating tumor cells (aCTC) in metastatic breast cancer. Breast cancer research and treatment. 2016;160(2):277-90.
18. De Luca F, Rotunno G, Salvianti F, Galardi F, Pestrin M, Gabellini S, et al. Mutational analysis of single circulating tumor cells by next generation sequencing in metastatic breast cancer. Oncotarget. 2016;7(18):26107-19.
19. Bulfoni M, Gerratana L, Del Ben F, Marzinotto S, Sorrentino M, Turetta M, et al. In patients with metastatic breast cancer the identification of circulating tumor cells in epithelial-to-mesenchymal transition is associated with a poor prognosis. Breast cancer research: BCR. 2016;18(1):30.
20. Brown-Glaberman U, Marron M, Chalasani P, Livingston R, Iannone M, Specht J, et al. Circulating Carbonic Anhydrase IX and Antiangiogenic Therapy in Breast Cancer. Disease markers. 2016;2016:9810383.
21. Bredemeier M, Edimiris P, Tewes M, Mach P, Aktas B, Schellbach D, et al. Establishment of a multimarker qPCR panel for the molecular characterization of circulating tumor cells in blood samples of metastatic breast cancer patients during the course of palliative treatment. Oncotarget. 2016;7(27):41677-90.
22. Bednarz-Knoll N, Efstathiou A, Gotzhein F, Wikman H, Mueller V, Kang Y, et al. Potential Involvement of Jagged1 in Metastatic Progression of Human Breast Carcinomas. Clinical chemistry. 2016;62(2):378-86.
23. Aktas B, Kasimir-Bauer S, Muller V, Janni W, Fehm T, Wallwiener D, et al. Comparison of the HER2, estrogen and progesterone receptor expression profile of primary tumor, metastases and circulating tumor cells in metastatic breast cancer patients. BMC cancer. 2016;16.
24. Zhang H, Luo M, Jin Z, Wang D, Sun M, Zhao X, et al. Expression and clinicopathological significance of FSIP1 in breast cancer. Oncotarget. 2015;6(12):10658-66.
25. Wang HY, Ahn S, Kim S, Park S, Jung D, Park S, et al. Detection of circulating tumor cell-specific markers in breast cancer patients using the quantitative RT-PCR assay. International journal of clinical oncology. 2015;20(5):878-90.
26. Wang D, Liu X, Hsieh B, Bruce R, Somlo G, Huang J, et al. Exploring Glycan Markers for Immunotyping and Precision-targeting of Breast Circulating Tumor Cells. Archives of medical research. 2015;46(8):642-50.
27. Wallwiener M, Hartkopf AD, Riethdorf S, Nees J, Sprick MR, Schonfisch B, et al. The impact of HER2 phenotype of circulating tumor cells in metastatic breast cancer: a retrospective study in 107 patients. BMC cancer. 2015;15:403.
28. Vishnoi M, Peddibhotla S, Yin W, Scamardo, AT, George GC, Hong DS, et al. The isolation and characterization of CTC subsets related to breast cancer dormancy. Scientific reports. 2015;5:17533.
29. Staudigl C, Concin N, Grimm C, Pfeiler G, Nehoda R, Singer CF, et al. Prognostic relevance of pretherapeutic gamma-glutamyltransferase in patients with primary metastatic breast cancer. PloS one. 2015;10(4):e0125317.
30. Shen B, Zheng MQ, Xu XY, Wei D. Translational Medicine Study on Circulating Tumor Cell Detection in Patients with Metastatic Breast Cancer. Journal of International Translational Medicine. 2015;3(2):93-7.
31. Shaker O, Maher M, Nassar Y, Morcos G, Gad Z. Role of microRNAs -29b-2, -155, -197 and -205 as diagnostic biomarkers in serum of breast cancer females. Gene. 2015;560(1):77-82.
32. Sefrioui D, Perdrix A, Sarafan-Vasseur N, Dolfus C, Dujon A, Picquenot JM, et al. Short report: Monitoring ESR1 mutations by circulating tumor DNA in aromatase inhibitor resistant metastatic breast cancer. International journal of cancer. 2015;137(10):2513-9.
33. Schneck H, Gierke B, Uppenkamp F, Behrens B, Niederacher D, Stoecklein NH, et al. EpCAM-Independent Enrichment of Circulating Tumor Cells in Metastatic Breast Cancer. PloS one. 2015;10(12):e0144535.
34. Satelli A, Brownlee Z, Mitra A, Meng QH, Li S. Circulating tumor cell enumeration with a combination of epithelial cell adhesion molecule- and cell-surface vimentin-based methods for monitoring breast cancer therapeutic response. Clinical chemistry. 2015;61(1):259-66.
35. Sarioglu AF, Aceto N, Kojic N, Donaldson MC, Zeinali M, Hamza B, et al. A microfluidic device for label-free, physical capture of circulating tumor cell clusters. Nature methods. 2015;12(7):685-91.
36. Polioudaki H, Agelaki S, Chiotaki R, Politaki E, Mavroudis D, Matikas A, et al. Variable expression levels of keratin and vimentin reveal differential EMT status of circulating tumor cells and correlation with clinical characteristics and outcome of patients with metastatic breast cancer. BMC cancer. 2015;15.
37. Pestrin M, Salvianti F, Galardi F, De Luca F, Turner N, Malorni L, et al. Heterogeneity of PIK3CA mutational status at the single cell level in circulating tumor cells from metastatic breast cancer patients. Molecular oncology. 2015;9(4):749-57.
38. Paoletti C, Muniz MC, Thomas DG, Griffith KA, Kidwell KM, Tokudome N, et al. Development of circulating tumor cell-endocrine therapy index in patients with hormone receptor-positive breast cancer. Clinical cancer research: An official journal of the American Association for Cancer Research. 2015;21(11):2487-98.
39. Paoletti C, Li Y, Muniz MC, Kidwell KM, Aung K, Thomas DG, et al. Significance of Circulating Tumor Cells in Metastatic Triple-Negative Breast Cancer Patients within a Randomized, Phase II Trial: TBCRC 019. Clinical cancer research: An official journal of the American Association for Cancer Research. 2015;21(12):2771-9.
40. Onstenk W, Sieuwerts AM, Weekhout M, Mostert B, Reijm EA, van Deurzen CH, et al. Gene expression profiles of circulating tumor cells versus primary tumors in metastatic breast cancer. Cancer letters. 2015;362(1):36-44.
41. Mego M, Zuo Z, Gao H, Cohen EN, Giordano A, Tin S, et al. Circulating tumour cells are linked to plasma D-dimer levels in patients with metastatic breast cancer. Thrombosis and haemostasis. 2015;113(3):593-8.
42. Maroni P, Bendinelli P, Morelli D, Drago L, Luzzati A, Perrucchini G, et al. High SPARC Expression Starting from Dysplasia, Associated with Breast Carcinoma, Is Predictive for Bone Metastasis without Enhancement of Plasma Levels. International journal of molecular sciences. 2015;16(12):28108-22.
43. Magbanua MJ, Carey LA, DeLuca A, Hwang J, Scott JH, Rimawi MF, et al. Circulating tumor cell analysis in metastatic triple-negative breast cancers. Clinical cancer research: An official journal of the American Association for Cancer Research. 2015;21(5):1098-105.
44. Madic J, Kiialainen A, Bidard FC, Birzele F, Ramey G, Leroy Q, et al. Circulating tumor DNA and circulating tumor cells in metastatic triple negative breast cancer patients. International journal of cancer. 2015;136(9):2158-65.
45. Lang JE, Scott JH, Wolf DM, Novak P, Punj V, Magbanua MJ, et al. Expression profiling of circulating tumor cells in metastatic breast cancer. Breast cancer research and treatment. 2015;149(1):121-31.
46. Kristiansen S, Jorgensen LM, Hansen MH, Nielsen D, Soletormos G. Concordance of Hypermethylated DNA and the Tumor Markers CA 15-3, CEA, and TPA in Serum during Monitoring of Patients with Advanced Breast Cancer. BioMed research international. 2015;2015:986024.
47. Kallergi G, Agelaki S, Papadaki MA, Nasias D, Matikas A, Mavroudis D, et al. Expression of truncated human epidermal growth factor receptor 2 on circulating tumor cells of breast cancer patients. Breast cancer research: BCR. 2015;17:113.
48. Kalinsky K, Mayer JA, Xu X, Pham T, Wong KL, Villarin E, et al. Correlation of hormone receptor status between circulating tumor cells, primary tumor, and metastasis in breast cancer patients. Clinical & translational oncology: official publication of the Federation of Spanish Oncology Societies and of the National Cancer Institute of Mexico. 2015;17(7):539-46.
49. Helissey C, Berger F, Cottu P, Dieras V, Mignot L, Servois V, et al. Circulating tumor cell thresholds and survival scores in advanced metastatic breast cancer: the observational step of the CirCe01 phase III trial. Cancer letters. 2015;360(2):213-8.
50. Guttery DS, Page K, Hills A, Woodley L, Marchese SD, Rghebi B, et al. Noninvasive detection of activating estrogen receptor 1 (ESR1) mutations in estrogen receptor-positive metastatic breast cancer. Clinical chemistry. 2015;61(7):974-82.
51. Guo M, Li X, Zhang S, Song H, Zhang W, Shang X, et al. Real-time quantitative RT-PCR detection of circulating tumor cells from breast cancer patients. International journal of oncology. 2015;46(1):281-9.
52. Gasch C, Plummer PN, Jovanovic L, McInnes LM, Wescott D, Saunders CM, et al. Heterogeneity of miR-10b expression in circulating tumor cells. Scientific reports. 2015;5:15980.
53. Frithiof H, Welinder C, Larsson AM, Ryden L, Aaltonen K. A novel method for downstream characterization of breast cancer circulating tumor cells following CellSearch isolation. Journal of translational medicine. 2015;13:126.
54. Fina E, Reduzzi C, Motta R, Di Cosimo S, Bianchi G, Martinetti A, et al. Did circulating tumor cells tell us all they could? The missed circulating tumor cell message in breast cancer. The International journal of biological markers. 2015;30(4):e429-33.
55. Fejzic H, Mujagic S, Azabagic S, Burina M. Tumor marker CA 15-3 in breast cancer patients. Acta medica academica. 2015;44(1):39-46.
56. Chen YG, Janckila A, Chao TY, Yeh RH, Gao HW, Lee SH, et al. Association of Tartrate-Resistant Acid Phosphatase-Expressed Macrophages and Metastatic Breast Cancer Progression. Medicine. 2015;94(48):e2165.
57. Ao Z, Shah SH, Machlin LM, Parajuli R, Miller PC, Rawal S, et al. Identification of Cancer-Associated Fibroblasts in Circulating Blood from Patients with Metastatic Breast Cancer. Cancer research. 2015;75(22):4681-7.
58. Antolin S, Calvo L, Blanco-Calvo M, Santiago MP, Lorenzo-Patino MJ, Haz-Conde M, et al. Circulating miR-200c and miR-141 and outcomes in patients with breast cancer. BMC cancer. 2015;15:297.
59. Agelaki S, Kalykaki A, Markomanolaki H, Papadaki MA, Kallergi G, Hatzidaki D, et al. Efficacy of Lapatinib in Therapy-Resistant HER2-Positive Circulating Tumor Cells in Metastatic Breast Cancer. PloS one. 2015;10(6):e0123683.
60. Xie S, Ding X, Mo W, Chen J. Serum tissue polypeptide-specific antigen is an independent predictor in breast cancer. Acta histochemica. 2014;116(2):372-6.
61. Williams A, Chung J, Ou X, Zheng G, Rawal S, Ao Z, et al. Fourier ptychographic microscopy for filtration-based circulating tumor cell enumeration and analysis. Journal of biomedical optics. 2014;19(6):066007.
62. Watanabe M, Uehara Y, Yamashita N, Fujimura Y, Nishio K, Sawada T, et al. Multicolor detection of rare tumor cells in blood using a novel flow cytometry-based system. Cytometry Part A: the journal of the International Society for Analytical Cytology. 2014;85(3):206-13.
63. Warkiani ME, Khoo BL, Tan DS, Bhagat AA, Lim WT, Yap YS, et al. An ultra-high-throughput spiral microfluidic biochip for the enrichment of circulating tumor cells. The Analyst. 2014;139(13):3245-55.
64. Wallwiener M, Riethdorf S, Hartkopf AD, Modugno C, Nees J, Madhavan D, et al. Serial enumeration of circulating tumor cells predicts treatment response and prognosis in metastatic breast cancer: a prospective study in 393 patients. BMC cancer. 2014;14:512.
65. Usiakova Z, Mikulova V, Pinterova D, Brychta M, Valchar J, Kubecova M, et al. Circulating tumor cells in patients with breast cancer: monitoring chemotherapy success. In vivo (Athens, Greece). 2014;28(4):605-14.
66. Tastekin D, Tas F, Karabulut S, Duranyildiz D, Serilmez M, Guveli M, et al. Clinical significance of serum tenascin-C levels in breast cancer. Tumour biology: the journal of the International Society for Oncodevelopmental Biology and Medicine. 2014;35(7):6619-25.
67. Stebbing J, Harding V, Urch CE, Kaier T, Schofield G, Flook M, et al. The prognostic role of circulating tumor cells in heavily pretreated individuals with a low life expectancy. Future oncology (London, England). 2014;10(16):2555-60.
68. Smerage JB, Barlow WE, Hortobagyi GN, Winer EP, Leyland-Jones B, Srkalovic G, et al. Circulating tumor cells and response to chemotherapy in metastatic breast cancer: SWOG S0500. Journal of clinical oncology: official journal of the American Society of Clinical Oncology. 2014;32(31):3483-9.
69. Shao X, Wang X, Xu X, Feng J, Han M, Zhang H, et al. Outcome prediction values of soluble human epidermal growth factor receptor-2 extracellular domain in metastatic breast cancer. International journal of clinical and experimental pathology. 2014;7(3):1108-13.
70. Santos MF, Mannam VK, Craft BS, Puneky LV, Sheehan NT, Lewis RE, et al. Comparative analysis of innate immune system function in metastatic breast, colorectal, and prostate cancer patients with circulating tumor cells. Experimental and molecular pathology. 2014;96(3):367-74.
71. Rothe F, Laes JF, Lambrechts D, Smeets D, Vincent D, Maetens M, et al. Plasma circulating tumor DNA as an alternative to metastatic biopsies for mutational analysis in breast cancer. Annals of oncology: official journal of the European Society for Medical Oncology/ESMO. 2014;25(10):1959-65.
72. Ramirez JM, Fehm T, Orsini M, Cayrefourcq L, Maudelonde T, Pantel K, et al. Prognostic relevance of viable circulating tumor cells detected by EPISPOT in metastatic breast cancer patients. Clinical chemistry. 2014;60(1):214-21.
73. Peeters DJ, van Dam PJ, Van den Eynden GG, Rutten A, Wuyts H, Pouillon L, et al. Detection and prognostic significance of circulating tumour cells in patients with metastatic breast cancer according to immunohistochemical subtypes. British journal of cancer. 2014;110(2):375-83.
74. Park IH, Kang JH, Lee KS, Nam S, Ro J, Kim JH. Identification and clinical implications of circulating microRNAs for estrogen receptor-positive breast cancer. Tumour biology: the journal of the International Society for Oncodevelopmental Biology and Medicine. 2014;35(12):12173-80.
75. Papadaki MA, Kallergi G, Zafeiriou Z, Manouras L, Theodoropoulos PA, Mavroudis D, et al. Co-expression of putative stemness and epithelial-to-mesenchymal transition markers on single circulating tumour cells from patients with early and metastatic breast cancer. BMC cancer. 2014;14:651.
76. Neves RP, Raba K, Schmidt O, Honisch E, Meier-Stiegen F, Behrens B, et al. Genomic high-resolution profiling of single CKpos/CD45neg flow-sorting purified circulating tumor cells from patients with metastatic breast cancer. Clinical chemistry. 2014;60(10):1290-7.
77. Miller KD, Althouse SK, Nabell L, Rugo H, Carey L, Kimmick G, et al. A phase II study of medroxyprogesterone acetate in patients with hormone receptor negative metastatic breast cancer: translational breast cancer research consortium trial 007. Breast cancer research and treatment. 2014;148(1):99-106.
78. Massarweh S, Moss J, Wang C, Romond E, Slone S, Weiss H, et al. Impact of adding the multikinase inhibitor sorafenib to endocrine therapy in metastatic estrogen receptor-positive breast cancer. Future oncology (London, England). 2014;10(15):2435-48.
79. Madhavan D, Wallwiener M, Bents K, Zucknick M, Nees J, Schott S, et al. Plasma DNA integrity as a biomarker for primary and metastatic breast cancer and potential marker for early diagnosis. Breast cancer research and treatment. 2014;146(1):163-74.
80. Lustberg MB, Balasubramanian P, Miller B, Garcia-Villa A, Deighan C, Wu Y, et al. Heterogeneous atypical cell populations are present in blood of metastatic breast cancer patients. Breast cancer research: BCR. 2014;16(2):R23.
81. Khoo BL, Warkiani ME, Tan DS, Bhagat AA, Irwin D, Lau DP, et al. Clinical validation of an ultra high-throughput spiral microfluidics for the detection and enrichment of viable circulating tumor cells. PloS one. 2014;9(7):e99409.
82. Kalykaki A, Agelaki S, Kallergi G, Xyrafas A, Mavroudis D, Georgoulias V. Elimination of EGFR-expressing circulating tumor cells in patients with metastatic breast cancer treated with gefitinib. Cancer chemotherapy and pharmacology. 2014;73(4):685-93.
83. Ignatiadis M, Riethdorf S, Bidard FC, Vaucher I, Khazour M, Rothe F, et al. International study on inter-reader variability for circulating tumor cells in breast cancer. Breast cancer research: BCR. 2014;16(2):R43.
84. Horn P, Jakobsen EH, Madsen JS, Brandslund I. New Approach for Interpreting Changes in Circulating Tumour Cells (CTC) for Evaluation of Treatment Effect in Metastatic Breast Cancer. Translational Oncology. 2014;7(6):694-701.
85. Heidary M, Auer M, Ulz P, Heitzer E, Petru E, Gasch C, et al. The dynamic range of circulating tumor DNA in metastatic breast cancer. Breast cancer research: BCR. 2014;16(4):421.
86. Hartkopf AD, Stefanescu D, Wallwiener M, Hahn M, Becker S, Solomayer EF, et al. Tumor cell dissemination to the bone marrow and blood is associated with poor outcome in patients with metastatic breast cancer. Breast cancer research and treatment. 2014;147(2):345-51.
87. Giuliano M, Giordano A, Jackson S, De Giorgi U, Mego M, Cohen EN, et al. Circulating tumor cells as early predictors of metastatic spread in breast cancer patients with limited metastatic dissemination. Breast cancer research: BCR. 2014;16(5):440.
88. Georgoulias V, Apostolaki S, Bozionelou V, Politaki E, Perraki M, Georgoulia N, et al. Effect of front-line chemotherapy on circulating CK-19 mRNA-positive cells in patients with metastatic breast cancer. Cancer chemotherapy and pharmacology. 2014;74(6):1217-25.
89. Gaykema SB, Schroder CP, Vitfell-Rasmussen J, Chua S, Oude Munnink TH, Brouwers AH, et al. 89Zr-trastuzumab and 89Zr-bevacizumab PET to evaluate the effect of the HSP90 inhibitor NVP-AUY922 in metastatic breast cancer patients. Clinical cancer research: an official journal of the American Association for Cancer Research. 2014;20(15):3945-54.
90. Galletti G, Sung MS, Vahdat LT, Shah MA, Santana SM, Altavilla G, et al. Isolation of breast cancer and gastric cancer circulating tumor cells by use of an anti HER2-based microfluidic device. Lab on a chip. 2014;14(1):147-56.
91. Fan M, Zhang J, Wang Z, Wang B, Zhang Q, Zheng C, et al. Phosphorylated VEGFR2 and hypertension: potential biomarkers to indicate VEGF-dependency of advanced breast cancer in anti-angiogenic therapy. Breast cancer research and treatment. 2014;143(1):141-51.
92. Fackler MJ, Lopez Bujanda Z, Umbricht C, Teo WW, Cho S, Zhang Z, et al. Novel methylated biomarkers and a robust assay to detect circulating tumor DNA in metastatic breast cancer. Cancer research. 2014;74(8):2160-70.
93. El-mezayen HA, Metwally FM, Darwish H. A novel discriminant score based on tumor-associated trypsin inhibitor for accurate diagnosis of metastasis in patients with breast cancer. Tumour biology: the journal of the International Society for Oncodevelopmental Biology and Medicine. 2014;35(3):2759-67.
94. Deng G, Krishnakumar S, Powell AA, Zhang H, Mindrinos MN, Telli ML, et al. Single cell mutational analysis of PIK3CA in circulating tumor cells and metastases in breast cancer reveals heterogeneity, discordance, and mutation persistence in cultured disseminated tumor cells from bone marrow. BMC cancer. 2014;14:456.
95. Chaari M, Ayadi I, Rousseau A, Lefkou E, Van Dreden P, Sidibe F, et al. Impact of breast cancer stage, time from diagnosis and chemotherapy on plasma and cellular biomarkers of hypercoagulability. BMC cancer. 2014;14:991.
96. Cao J, Zhang J, Wang Z, Wang B, Lv F, Wang L, et al. Hypothyroidism as a potential biomarker of efficacy of famitinib, a novel VEGFR-2 inhibitor in metastatic breast cancer. Cancer chemotherapy and pharmacology. 2014;74(2):389-98.
97. Bock C, Rack B, Huober J, Andergassen U, Jeschke U, Doisneau-Sixou S. Distinct expression of cytokeratin, N-cadherin and CD133 in circulating tumor cells of metastatic breast cancer patients. Future oncology (London, England). 2014;10(10):1751-65.
98. Bidard FC, Peeters DJ, Fehm T, Nole F, Gisbert-Criado R, Mavroudis D, et al. Clinical validity of circulating tumour cells in patients with metastatic breast cancer: a pooled analysis of individual patient data. The Lancet Oncology. 2014;15(4):406-14.
99. Baskic D, Popovic S, Bankovic D, Arsovic A, Vukovic V, Zelen I, et al. Evaluation of inflammatory biomarkers as helping diagnostic tool in patients with breast cancer. Cancer biomarkers: section A of Disease markers. 2014;14(6):401-8.
100. Baselga J, Cortes J, Im SA, Clark E, Ross G, Kiermaier A, et al. Biomarker analyses in CLEOPATRA: a phase III, placebo-controlled study of pertuzumab in human epidermal growth factor receptor 2-positive, first-line metastatic breast cancer. Journal of clinical oncology: official journal of the American Society of Clinical Oncology. 2014;32(33):3753-61.
101. Anfossi S, Giordano A, Gao H, Cohen EN, Tin S, Wu Q, et al. High serum miR-19a levels are associated with inflammatory breast cancer and are predictive of favorable clinical outcome in patients with metastatic HER2+ inflammatory breast cancer. PloS one. 2014;9(1):e83113.
102. Zhao S, Yang H, Zhang M, Zhang D, Liu Y, Liu Y, et al. Circulating tumor cells (CTCs) detected by triple-marker EpCAM, CK19, and hMAM RT-PCR and their relation to clinical outcome in metastatic breast cancer patients. Cell biochemistry and biophysics. 2013;65(2):263-73.
103. Zhao LN, Li PF, Li FD, Yang YK, Liu N, Cai L. The prognostic value of circulating tumor cells lacking cytokeratins in metastatic breast cancer patients. Journal of cancer research and therapeutics. 2013;9(1):29-37.
104. Wu Y, Deighan CJ, Miller BL, Balasubramanian P, Lustberg MB, Zborowski M, et al. Isolation and analysis of rare cells in the blood of cancer patients using a negative depletion methodology. Methods (San Diego, Calif). 2013;64(2):169-82.
105. Wallwiener M, Hartkopf AD, Baccelli I, Riethdorf S, Schott S, Pantel K, et al. The prognostic impact of circulating tumor cells in subtypes of metastatic breast cancer. Breast cancer research and treatment. 2013;137(2):503-10.
106. Turker I, Uyeturk U, Sonmez OU, Oksuzoglu B, Helvaci K, Arslan UY, et al. Detection of circulating tumor cells in breast cancer patients: prognostic predictive role. Asian Pacific journal of cancer prevention: APJCP. 2013;14(3):1601-7.
107. Tryfonidis K, Boukovinas I, Xenidis N, Christophyllakis C, Papakotoulas P, Politaki E, et al. A multicenter phase I-II study of docetaxel plus epirubicin plus bevacizumab as first-line treatment in women with HER2-negative metastatic breast cancer. Breast (Edinburgh, Scotland). 2013;22(6):1171-7.
108. Tarhan MO, Gonel A, Kucukzeybek Y, Erten C, Cuhadar S, Yigit SC, et al. Prognostic significance of circulating tumor cells and serum CA15-3 levels in metastatic breast cancer, single center experience, preliminary results. Asian Pacific journal of cancer prevention: APJCP. 2013;14(3):1725-9.
109. Strati A, Kasimir-Bauer S, Markou A, Parisi C, Lianidou ES. Comparison of three molecular assays for the detection and molecular characterization of circulating tumor cells in breast cancer. Breast cancer research: BCR. 2013;15(2):R20.
110. Stoetzer OJ, Fersching DM, Salat C, Steinkohl O, Gabka CJ, Hamann U, et al. Prediction of response to neoadjuvant chemotherapy in breast cancer patients by circulating apoptotic biomarkers nucleosomes, DNAse, cytokeratin-18 fragments and survivin. Cancer letters. 2013;336(1):140-8.
111. Stoetzer OJ, Fersching DM, Salat C, Steinkohl O, Gabka CJ, Hamann U, et al. Circulating immunogenic cell death biomarkers HMGB1 and RAGE in breast cancer patients during neoadjuvant chemotherapy. Tumour biology: the journal of the International Society for Oncodevelopmental Biology and Medicine. 2013;34(1):81-90.
112. Stebbing J, Payne R, Reise J, Frampton AE, Avery M, Woodley L, et al. The Efficacy of Lapatinib in Metastatic Breast Cancer with HER2 Non-Amplified Primary Tumors and EGFR Positive Circulating Tumor Cells: A Proof-Of-Concept Study. PloS one. 2013;8(5).
113. Song G, Wang X, Jia J, Yuan Y, Wan F, Zhou X, et al. Elevated level of peripheral CD8(+)CD28(−) T lymphocytes are an independent predictor of progression-free survival in patients with metastatic breast cancer during the course of chemotherapy. Cancer immunology, immunotherapy: CII. 2013;62(6):1123-30.
114. Smerage JB, Budd GT, Doyle GV, Brown M, Paoletti C, Muniz M, et al. Monitoring apoptosis and Bcl-2 on circulating tumor cells in patients with metastatic breast cancer. Molecular oncology. 2013;7(3):680-92.
115. Schneck H, Blassl C, Meier-Stiegen F, Neves RP, Janni W, Fehm T, et al. Analysing the mutational status of PIK3CA in circulating tumor cells from metastatic breast cancer patients. Molecular oncology. 2013;7(5):976-86.
116. Schindlbeck C, Andergassen U, Hofmann S, Juckstock J, Jeschke U, Sommer H, et al. Comparison of circulating tumor cells (CTC) in peripheral blood and disseminated tumor cells in the bone marrow (DTC-BM) of breast cancer patients. Journal of cancer research and clinical oncology. 2013;139(6):1055-62.
117. Roop RP, Naughton MJ, Van Poznak C, Schneider JG, Lammers PE, Pluard TJ, et al. A randomized phase II trial investigating the effect of platelet function inhibition on circulating tumor cells in patients with metastatic breast cancer. Clinical breast cancer. 2013;13(6):409-15.
118. Perroud HA, Rico MJ, Alasino CM, Pezzotto SM, Rozados VR, Scharovsky OG. Association between baseline VEGF/sVEGFR-2 and VEGF/TSP-1 ratios and response to metronomic chemotherapy using cyclophosphamide and celecoxib in patients with advanced breast cancer. Indian journal of cancer. 2013;50(2):115-21.
119. Panis C, Herrera AC, Victorino VJ, Aranome AM, Cecchini R. Screening of circulating TGF-beta levels and its clinicopathological significance in human breast cancer. Anticancer research. 2013;33(2):737-42.
120. Palmieri C, Caley MP, Purshouse K, Fonseca AV, Rodriguez-Teja M, Kogianni G, et al. Endo180 modulation by bisphosphonates and diagnostic accuracy in metastatic breast cancer. British journal of cancer. 2013;108(1):163-9.
121. Miles DW, de Haas SL, Dirix LY, Romieu G, Chan A, Pivot X, et al. Biomarker results from the AVADO phase 3 trial of first-line bevacizumab plus docetaxel for HER2-negative metastatic breast cancer. British journal of cancer. 2013;108(5):1052-60.
122. Martin M, Custodio S, de las Casas MLM, Garcia-Saenz JA, de la Torre JC, Bellon-Cano JM, et al. Circulating Tumor Cells Following First Chemotherapy Cycle: An Early and Strong Predictor of Outcome in Patients With Metastatic Breast Cancer. The oncologist. 2013;18(8):917-23.
123. Magbanua MJ, Sosa EV, Roy R, Eisenbud LE, Scott JH, Olshen A, et al. Genomic profiling of isolated circulating tumor cells from metastatic breast cancer patients. Cancer research. 2013;73(1):30-40.
124. Liu Y, Liu Q, Wang T, Bian L, Zhang S, Hu H, et al. Circulating tumor cells in HER2-positive metastatic breast cancer patients: a valuable prognostic and predictive biomarker. BMC cancer. 2013;13:202.
125. Ligthart ST, Bidard FC, Decraene C, Bachelot T, Delaloge S, Brain E, et al. Unbiased quantitative assessment of Her-2 expression of circulating tumor cells in patients with metastatic and non-metastatic breast cancer. Annals of Oncology. 2013;24(5):1231-8.
126. Lasa A, Garcia A, Alonso C, Millet P, Cornet M, Ramon y Cajal T, et al. Molecular detection of peripheral blood breast cancer mRNA transcripts as a surrogate biomarker for circulating tumor cells. PloS one. 2013;8(9):e74079.
127. Kontani K, Kuroda N, Hashimoto S, Murazawa C, Norimura S, Tanaka H, et al. Clinical usefulness of human epidermal growth factor receptor-2 extracellular domain as a biomarker for monitoring cancer status and predicting the therapeutic efficacy in breast cancer. Cancer biology & therapy. 2013;14(1):20-8.
128. Kallergi G, Konstantinidis G, Markomanolaki H, Papadaki MA, Mavroudis D, Stournaras C, et al. Apoptotic circulating tumor cells in early and metastatic breast cancer patients. Molecular cancer therapeutics. 2013;12(9):1886-95.
129. Jiang ZF, Cristofanilli M, Shao ZM, Tong ZS, Song EW, Wang XJ, et al. Circulating tumor cells predict progression-free and overall survival in Chinese patients with metastatic breast cancer, HER2-positive or triple-negative (CBCSG004): a multicenter, double-blind, prospective trial. Annals of oncology: official journal of the European Society for Medical Oncology/ESMO. 2013;24(11):2766-72.
130. Hyun KA, Kwon K, Han H, Kim SI, Jung HI. Microfluidic flow fractionation device for label-free isolation of circulating tumor cells (CTCs) from breast cancer patients. Biosensors & bioelectronics. 2013;40(1):206-12.
131. Green TL, Cruse JM, Lewis RE, Craft BS. Circulating tumor cells (CTCs) from metastatic breast cancer patients linked to decreased immune function and response to treatment. Experimental and molecular pathology. 2013;95(2):174-9.
132. El-Mezayen HA, Toson el SA, Darwish H, Metwally FM. Development of a novel metastatic breast cancer score based on hyaluronic acid metabolism. Medical oncology (Northwood, London, England). 2013;30(1):404.
133. Eichelser C, Flesch-Janys D, Chang-Claude J, Pantel K, Schwarzenbach H. Deregulated serum concentrations of circulating cell-free microRNAs miR-17, miR-34a, miR-155, and miR-373 in human breast cancer development and progression. Clinical chemistry. 2013;59(10):1489-96.
134. Divella R, Daniele A, Savino E, Palma F, Bellizzi A, Giotta F, et al. Circulating levels of transforming growth factor-betaeta (TGF-beta) and chemokine (C-X-C motif) ligand-1 (CXCL1) as predictors of distant seeding of circulating tumor cells in patients with metastatic breast cancer. Anticancer research. 2013;33(4):1491-7.
135. Dawson SJ, Tsui DW, Murtaza M, Biggs H, Rueda OM, Chin SF, et al. Analysis of circulating tumor DNA to monitor metastatic breast cancer. The New England journal of medicine. 2013;368(13):1199-209.
136. Coumans FA, van Dalum G, Beck M, Terstappen LW. Filter characteristics influencing circulating tumor cell enrichment from whole blood. PloS one. 2013;8(4):e61770.
137. Bjohle J, Bergqvist J, Gronowitz JS, Johansson H, Carlsson L, Einbeigi Z, et al. Serum thymidine kinase activity compared with CA 15-3 in locally advanced and metastatic breast cancer within a randomized trial. Breast cancer research and treatment. 2013;139(3):751-8.
138. Bechmann T, Andersen RF, Pallisgaard N, Madsen JS, Maae E, Jakobsen EH, et al. Plasma HER2 amplification in cell-free DNA during neoadjuvant chemotherapy in breast cancer. Journal of cancer research and clinical oncology. 2013;139(6):995-1003.
139. Baccelli I, Schneeweiss A, Riethdorf S, Stenzinger A, Schillert A, Vogel V, et al. Identification of a population of blood circulating tumor cells from breast cancer patients that initiates metastasis in a xenograft assay. Nature biotechnology. 2013;31(6):539-44.
140. Babayan A, Hannemann J, Spotter J, Muller V, Pantel K, Joosse SA. Heterogeneity of estrogen receptor expression in circulating tumor cells from metastatic breast cancer patients. PloS one. 2013;8(9):e75038.
141. Andergassen U, Hofmann S, Kolbl AC, Schindlbeck C, Neugebauer J, Hutter S, et al. Detection of tumor cell-specific mRNA in the peripheral blood of patients with breast cancer-evaluation of several markers with real-time reverse transcription-PCR. International journal of molecular sciences. 2013;14(1):1093-104.
142. Aktas B, Kasimir-Bauer S, Lehmann N, Kimmig R, Tewes M. Validity of bone marker measurements for monitoring response to bisphosphonate therapy with zoledronic acid in metastatic breast cancer. Oncology reports. 2013;30(1):441-7.
143. Zhao FL, Hu GD, Wang XF, Zhang XH, Zhang YK, Yu ZS. Serum overexpression of microRNA-10b in patients with bone metastatic primary breast cancer. The Journal of international medical research. 2012;40(3):859-66.
144. Yamamoto N, Nakayama T, Kajita M, Miyake T, Iwamoto T, Kim SJ, et al. Detection of aberrant promoter methylation of GSTP1, RASSF1A, and RARbeta2 in serum DNA of patients with breast cancer by a newly established one-step methylation-specific PCR assay. Breast cancer research and treatment. 2012;132(1):165-73.
145. Weissenstein U, Schumann A, Reif M, Link S, Toffol-Schmidt UD, Heusser P. Detection of circulating tumor cells in blood of metastatic breast cancer patients using a combination of cytokeratin and EpCAM antibodies. BMC cancer. 2012;12:206.
146. Vera-Ramirez L, Sanchez-Rovira P, Ramirez-Tortosa MC, Ramirez-Tortosa CL, Granados-Principal S, Lorente JA, et al. Oxidative stress status in metastatic breast cancer patients receiving palliative chemotherapy and its impact on survival rates. Free radical research. 2012;46(1):2-10.
147. Pierga JY, Hajage D, Bachelot T, Delaloge S, Brain E, Campone M, et al. High independent prognostic and predictive value of circulating tumor cells compared with serum tumor markers in a large prospective trial in first-line chemotherapy for metastatic breast cancer patients. Annals of oncology: official journal of the European Society for Medical Oncology/ESMO. 2012;23(3):618-24.
148. Pestrin M, Bessi S, Puglisi F, Minisini AM, Masci G, Battelli N, et al. Final results of a multicenter phase II clinical trial evaluating the activity of single-agent lapatinib in patients with HER2-negative metastatic breast cancer and HER2-positive circulating tumor cells. A proof-of-concept study. Breast cancer research and treatment. 2012;134(1):283-9.
149. Pectasides D, Papaxoinis G, Kotoula V, Fountzilas H, Korantzis I, Koutras A, et al. Expression of angiogenic markers in the peripheral blood of docetaxel-treated advanced breast cancer patients: a Hellenic Cooperative Oncology Group (HeCOG) study. Oncology reports. 2012;27(1):216-24.
150. Payne RE, Wang F, Su N, Krell J, Zebrowski A, Yague E, et al. Viable circulating tumour cell detection using multiplex RNA in situ hybridisation predicts progression-free survival in metastatic breast cancer patients. British journal of cancer. 2012;106(11):1790-7.
151. Munzone E, Botteri E, Sandri MT, Esposito A, Adamoli L, Zorzino L, et al. Prognostic value of circulating tumor cells according to immunohistochemically defined molecular subtypes in advanced breast cancer. Clinical breast cancer. 2012;12(5):340-6.
152. Montagna E, Cancello G, Bagnardi V, Pastrello D, Dellapasqua S, Perri G, et al. Metronomic chemotherapy combined with bevacizumab and erlotinib in patients with metastatic HER2-negative breast cancer: clinical and biological activity. Clinical breast cancer. 2012;12(3):207-14.
153. Mego M, Gao H, Lee BN, Cohen EN, Tin S, Giordano A, et al. Prognostic Value of EMT-Circulating Tumor Cells in Metastatic Breast Cancer Patients Undergoing High-Dose Chemotherapy with Autologous Hematopoietic Stem Cell Transplantation. Journal of Cancer. 2012;3:369-80.
154. Marrinucci D, Bethel K, Kolatkar A, Luttgen MS, Malchiodi M, Baehring F, et al. Fluid biopsy in patients with metastatic prostate, pancreatic and breast cancers. Physical biology. 2012;9(1):016003.
155. Madhavan D, Zucknick M, Wallwiener M, Cuk K, Modugno C, Scharpff M, et al. Circulating miRNAs as surrogate markers for circulating tumor cells and prognostic markers in metastatic breast cancer. Clinical cancer research: an official journal of the American Association for Cancer Research. 2012;18(21):5972-82.
156. Korantzis I, Kalogeras KT, Papaxoinis G, Kotoula V, Koutras A, Soupos N, et al. Expression of angiogenic markers in the peripheral blood of patients with advanced breast cancer treated with weekly docetaxel. Anticancer research. 2012;32(10):4569-80.
157. Keyvanjah K, DePrimo SE, Harmon CS, Huang X, Kern KA, Carley W. Soluble KIT correlates with clinical outcome in patients with metastatic breast cancer treated with sunitinib. Journal of translational medicine. 2012;10:165.
158. Joosse SA, Hannemann J, Spotter J, Bauche A, Andreas A, Muller V, et al. Changes in keratin expression during metastatic progression of breast cancer: impact on the detection of circulating tumor cells. Clinical cancer research: an official journal of the American Association for Cancer Research. 2012;18(4):993-1003.
159. Jain S, Ward MM, O’Loughlin J, Boeck M, Wiener N, Chuang E, et al. Incremental increase in VEGFR1(+) hematopoietic progenitor cells and VEGFR2(+) endothelial progenitor cells predicts relapse and lack of tumor response in breast cancer patients. Breast cancer research and treatment. 2012;132(1):235-42.
160. Higgins MJ, Jelovac D, Barnathan E, Blair B, Slater S, Powers P, et al. Detection of tumor PIK3CA status in metastatic breast cancer using peripheral blood. Clinical cancer research: an official journal of the American Association for Cancer Research. 2012;18(12):3462-9.
161. Hayashi N, Nakamura S, Tokuda Y, Shimoda Y, Yagata H, Yoshida A, et al. Prognostic value of HER2-positive circulating tumor cells in patients with metastatic breast cancer. International journal of clinical oncology. 2012;17(2):96-104.
162. Giordano A, Giuliano M, De Laurentiis M, Arpino G, Jackson S, Handy BC, et al. Circulating tumor cells in immunohistochemical subtypes of metastatic breast cancer: lack of prediction in HER2-positive disease treated with targeted therapy. Annals of Oncology. 2012;23(5):1144-50.
163. Giordano A, Gao H, Anfossi S, Cohen E, Mego M, Lee BN, et al. Epithelial-mesenchymal transition and stem cell markers in patients with HER2-positive metastatic breast cancer. Molecular cancer therapeutics. 2012;11(11):2526-34.
164. El-Arab LR, Swellam M, El Mahdy MM. Metronomic chemotherapy in metastatic breast cancer: impact on VEGF. Journal of the Egyptian National Cancer Institute. 2012;24(1):15-22.
165. De Giorgi U, Mego M, Scarpi E, Giuliano M, Giordano A, Reuben JM, et al. Relationship between lymphocytopenia and circulating tumor cells as prognostic factors for overall survival in metastatic breast cancer. Clinical breast cancer. 2012;12(4):264-9.
166. de Albuquerque A, Kaul S, Breier G, Krabisch P, Fersis N. Multimarker Analysis of Circulating Tumor Cells in Peripheral Blood of Metastatic Breast Cancer Patients: A Step Forward in Personalized Medicine. Breast Care. 2012;7(1):7-12.
167. Contractor K, Aboagye EO, Jacob J, Challapalli A, Coombes RC, Stebbing J. Monitoring early response to taxane therapy in advanced breast cancer with circulating tumor cells and [(18)F] 3-deoxy-3-fluorothymidine PET: a pilot study. Biomarkers in medicine. 2012;6(2):231-3.
168. Carlsson MC, Balog CI, Kilsgard O, Hellmark T, Bakoush O, Segelmark M, et al. Different fractions of human serum glycoproteins bind galectin-1 or galectin-8, and their ratio may provide a refined biomarker for pathophysiological conditions in cancer and inflammatory disease. Biochimica et biophysica acta. 2012;1820(9):1366-72.
169. Bordbar E, Malekzadeh M, Ardekani MT, Doroudchi M, Ghaderi A. Serum levels of G-CSF and IL-7 in Iranian breast cancer patients. Asian Pacific journal of cancer prevention: APJCP. 2012;13(10):5307-12.
170. Bock C, Rack B, Kuhn C, Hofmann S, Finkenzeller C, Jager B, et al. Heterogeneity of ER α and ErbB2 Status in Cell Lines and Circulating Tumor Cells of Metastatic Breast Cancer Patients. Translational Oncology. 2012;5(6):475-U172.
171. Bidard FC, Hajage D, Bachelot T, Delaloge S, Brain E, Campone M, et al. Assessment of circulating tumor cells and serum markers for progression-free survival prediction in metastatic breast cancer: a prospective observational study. Breast cancer research: BCR. 2012;14(1):R29.
172. Apostolou P, Toloudi M, Chatziioannou M, Ioannou E, Papasotiriou I. Cancer stem cells stemness transcription factors expression correlates with breast cancer disease stage. Current stem cell research & therapy. 2012;7(6):415-9.
173. Androulakis N, Agelaki S, Perraki M, Apostolaki S, Bozionelou V, Pallis A, et al. Clinical relevance of circulating CK-19mRNA-positive tumour cells before front-line treatment in patients with metastatic breast cancer. British journal of cancer. 2012;106(12):1917-25.
174. Andreopoulou E, Yang LY, Rangel KM, Reuben JM, Hsu L, Krishnamurthy S, et al. Comparison of assay methods for detection of circulating tumor cells in metastatic breast cancer: AdnaGen AdnaTest BreastCancer Select/Detect versus Veridex CellSearch system. International journal of cancer. 2012;130(7):1590-7.
175. Tuszynski GP, Rothman VL, Zheng X, Gutu M, Zhang X, Chang F. G-protein coupled receptor-associated sorting protein 1 (GASP-1), a potential biomarker in breast cancer. Experimental and molecular pathology. 2011;91(2):608-13.
176. Tokudome N, Ito Y, Takahashi S, Kobayashi K, Taira S, Tsutsumi C, et al. Detection of circulating tumor cells in peripheral blood of heavily treated metastatic breast cancer patients. Breast cancer (Tokyo, Japan). 2011;18(3):195-202.
177. Tao M, Ma D, Li Y, Zhou C, Li Y, Zhang Y, et al. Clinical significance of circulating tumor cells in breast cancer patients. Breast cancer research and treatment. 2011;129(1):247-54.
178. Tang JH, Zhao JH, Lu JW, Yan F, Qin JW, Xu B. Circulating levels of angiogenic cytokines in advanced breast cancer patients with system chemotherapy and their potential value in monitoring disease course. Journal of cancer research and clinical oncology. 2011;137(1):55-63.
179. Somlo G, Lau SK, Frankel P, Hsieh HB, Liu X, Yang L, et al. Multiple biomarker expression on circulating tumor cells in comparison to tumor tissues from primary and metastatic sites in patients with locally advanced/inflammatory, and stage IV breast cancer, using a novel detection technology. Breast cancer research and treatment. 2011;128(1):155-63.
180. Sieuwerts AM, Mostert B, Bolt-de Vries J, Peeters D, de Jongh FE, Stouthard JM, et al. mRNA and microRNA expression profiles in circulating tumor cells and primary tumors of metastatic breast cancer patients. Clinical cancer research: an official journal of the American Association for Cancer Research. 2011;17(11):3600-18.
181. Sanislo L, Vertakova-Krakovska B, Kuliffay P, Brtko J, Galbava A, Galbavy S. Detection of circulating tumor cells in metastatic breast cancer patients. Endocrine regulations. 2011;45(3):113-24.
182. Saldova R, Reuben JM, Abd Hamid UM, Rudd PM, Cristofanilli M. Levels of specific serum *N*-glycans identify breast cancer patients with higher circulating tumor cell counts. Annals of oncology: official journal of the European Society for Medical Oncology/ESMO. 2011;22(5):1113-9.
183. Reinholz MM, Kitzmann KA, Tenner K, Hillman D, Dueck AC, Hobday TJ, et al. Cytokeratin-19 and mammaglobin gene expression in circulating tumor cells from metastatic breast cancer patients enrolled in North Central Cancer Treatment Group trials, N0234/336/436/437. Clinical cancer research: an official journal of the American Association for Cancer Research. 2011;17(22):7183-93.
184. Purwanto I, Kurnianda J, Hariadi KW, Harijadi, Aryandono T, Setiaji K, et al. Concentration of serum HER-2/neu as a prognostic factor in locally advanced breast cancer (LABC) and metastatic breast cancer (MBC). Acta medica Indonesiana. 2011;43(1):23-8.
185. Pivetta E, Scapolan M, Pecolo M, Wassermann B, Abu-Rumeileh I, Balestreri L, et al. MMP-13 stimulates osteoclast differentiation and activation in tumour breast bone metastases. Breast cancer research: BCR. 2011;13(5):R105.
186. Peeters DJ, Van den Eynden GG, van Dam PJ, Prove A, Benoy IH, van Dam PA, et al. Circulating tumour cells in the central and the peripheral venous compartment in patients with metastatic breast cancer. British journal of cancer. 2011;104(9):1472-7.
187. Page K, Hava N, Ward B, Brown J, Guttery DS, Ruangpratheep C, et al. Detection of HER2 amplification in circulating free DNA in patients with breast cancer. British journal of cancer. 2011;104(8):1342-8.
188. Pachmann K, Camara O, Kohlhase A, Rabenstein C, Kroll T, Runnebaum IB, et al. Assessing the efficacy of targeted therapy using circulating epithelial tumor cells (CETC): the example of SERM therapy monitoring as a unique tool to individualize therapy. Journal of cancer research and clinical oncology. 2011;137(5):821-8.
189. Muller V, Riethdorf S, Rack B, Janni W, Fasching PA, Solomayer E, et al. Prospective evaluation of serum tissue inhibitor of metalloproteinase 1 and carbonic anhydrase IX in correlation to circulating tumor cells in patients with metastatic breast cancer. Breast cancer research: BCR. 2011;13(4):R71.
190. Mostert B, Kraan J, Bolt-de Vries J, van der Spoel P, Sieuwerts AM, Schutte M, et al. Detection of circulating tumor cells in breast cancer may improve through enrichment with anti-CD146. Breast cancer research and treatment. 2011;127(1):33-41.
191. Molloy TJ, Devriese LA, Helgason HH, Bosma AJ, Hauptmann M, Voest EE, et al. A multimarker QPCR-based platform for the detection of circulating tumour cells in patients with early-stage breast cancer. British journal of cancer. 2011;104(12):1913-9.
192. Mercatali L, Ibrahim T, Sacanna E, Flamini E, Scarpi E, Calistri D, et al. Bone metastases detection by circulating biomarkers: OPG and RANK-L. International journal of oncology. 2011;39(1):255-61.
193. Mego M, De Giorgi U, Dawood S, Wang X, Valero V, Andreopoulou E, et al. Characterization of metastatic breast cancer patients with nondetectable circulating tumor cells. International journal of cancer. 2011;129(2):417-23.
194. Mayer JA, Pham T, Wong KL, Scoggin J, Sales EV, Clarin T, et al. FISH-based determination of HER2 status in circulating tumor cells isolated with the microfluidic CEE platform. Cancer genetics. 2011;204(11):589-95.
195. Mayer EL, Dallabrida SM, Rupnick MA, Redline WM, Hannagan K, Ismail NS, et al. Contrary effects of the receptor tyrosine kinase inhibitor vandetanib on constitutive and flow-stimulated nitric oxide elaboration in humans. Hypertension. 2011;58(1):85-92.
196. Liska V, Holubec L, Jr., Treska V, Vrzalova J, Skalicky T, Sutnar A, et al. Evaluation of tumour markers as differential diagnostic tool in patients with suspicion of liver metastases from breast cancer. Anticancer research. 2011;31(4):1447-51.
197. Lipton A, Leitzel K, Ali SM, Carney W, Platek G, Steplewski K, et al. Human epidermal growth factor receptor 2 (HER2) extracellular domain levels are associated with progression-free survival in patients with HER2-positive metastatic breast cancer receiving lapatinib monotherapy. Cancer. 2011;117(21):5013-20.
198. Konigsberg R, Obermayr E, Bises G, Pfeiler G, Gneist M, Wrba F, et al. Detection of EpCAM positive and negative circulating tumor cells in metastatic breast cancer patients. Acta oncologica (Stockholm, Sweden). 2011;50(5):700-10.
199. Kim SJ, Masago A, Tamaki Y, Akazawa K, Tsukamoto F, Sato J, et al. A novel approach using telomerase-specific replication-selective adenovirus for detection of circulating tumor cells in breast cancer patients. Breast cancer research and treatment. 2011;128(3):765-73.
200. Kim P, Liu XJ, Lee T, Liu LM, Barham R, Kirkland R, et al. Highly sensitive proximity mediated immunoassay reveals HER2 status conversion in the circulating tumor cells of metastatic breast cancer patients. Proteome Science. 2011;9.
201. Kallergi G, Papadaki MA, Politaki E, Mavroudis D, Georgoulias V, Agelaki S. Epithelial to mesenchymal transition markers expressed in circulating tumour cells of early and metastatic breast cancer patients. Breast cancer research: BCR. 2011;13(3):R59.
202. Ignatiadis M, Rothe F, Chaboteaux C, Durbecq V, Rouas G, Criscitiello C, et al. HER2-positive circulating tumor cells in breast cancer. PloS one. 2011;6(1):e15624.
203. Hayashi N, Nakamura S, Yagata H, Shimoda Y, Ota H, Hortobagyi GN, et al. Chromosome 17 polysomy in circulating tumor cells in patients with metastatic breast cancer: a case series. International journal of clinical oncology. 2011;16(5):596-600.
204. Hartkopf AD, Wagner P, Wallwiener D, Fehm T, Rothmund R. Changing levels of circulating tumor cells in monitoring chemotherapy response in patients with metastatic breast cancer. Anticancer research. 2011;31(3):979-84.
205. Gradilone A, Raimondi C, Nicolazzo C, Petracca A, Gandini O, Vincenzi B, et al. Circulating tumour cells lacking cytokeratin in breast cancer: the importance of being mesenchymal. Journal of cellular and molecular medicine. 2011;15(5):1066-70.
206. Gradilone A, Raimondi C, Naso G, Silvestri I, Repetto L, Palazzo A, et al. How Circulating Tumor Cells Escape From Multidrug Resistance Translating Molecular Mechanisms in Metastatic Breast Cancer Treatment. American Journal of Clinical Oncology-Cancer Clinical Trials. 2011;34(6):625-7.
207. Gradilone A, Naso G, Raimondi C, Cortesi E, Gandini O, Vincenzi B, et al. Circulating tumor cells (CTCs) in metastatic breast cancer (MBC): prognosis, drug resistance and phenotypic characterization. Annals of oncology: official journal of the European Society for Medical Oncology/ESMO. 2011;22(1):86-92.
208. Giuliano M, Giordano A, Jackson S, Hess KR, De Giorgi U, Mego M, et al. Circulating tumor cells as prognostic and predictive markers in metastatic breast cancer patients receiving first-line systemic treatment. Breast cancer research: BCR. 2011;13(3):R67.
209. Etienne-Grimaldi MC, Formento P, Degeorges A, Pierga JY, Delva R, Pivot X, et al. Prospective analysis of the impact of VEGF-A gene polymorphisms on the pharmacodynamics of bevacizumab-based therapy in metastatic breast cancer patients. British journal of clinical pharmacology. 2011;71(6):921-8.
210. Consoli F, Grisanti S, Amoroso V, Almici C, Verardi R, Marini M, et al. Circulating tumor cells as predictors of prognosis in metastatic breast cancer: clinical application outside a clinical trial. Tumori. 2011;97(6):737-42.
211. Chimonidou M, Strati A, Tzitzira A, Sotiropoulou G, Malamos N, Georgoulias V, et al. DNA methylation of tumor suppressor and metastasis suppressor genes in circulating tumor cells. Clinical chemistry. 2011;57(8):1169-77.
212. Cheng JP, Yan Y, Wang XY, Lu YL, Yuan YH, Jia J, et al. MUC1-positive circulating tumor cells and MUC1 protein predict chemotherapeutic efficacy in the treatment of metastatic breast cancer. Chinese journal of cancer. 2011;30(1):54-61.
213. Breton F, Bennetau B, Lidereau R, Thomas L, Regnier G, Ehrhart JC, et al. A mesofluidic multiplex immunosensor for detection of circulating cytokeratin-positive cells in the blood of breast cancer patients. Biomedical microdevices. 2011;13(1):1-9.
214. Asaga S, Kuo C, Nguyen T, Terpenning M, Giuliano AE, Hoon DS. Direct serum assay for microRNA-21 concentrations in early and advanced breast cancer. Clinical chemistry. 2011;57(1):84-91.
215. Armstrong AJ, Marengo MS, Oltean S, Kemeny G, Bitting RL, Turnbull JD, et al. Circulating Tumor Cells from Patients with Advanced Prostate and Breast Cancer Display Both Epithelial and Mesenchymal Markers. Molecular Cancer Research. 2011;9(8):997-1007.
216. Appaiah HN, Goswami CP, Mina LA, Badve S, Sledge GW, Jr., Liu Y, et al. Persistent upregulation of U6:SNORD44 small RNA ratio in the serum of breast cancer patients. Breast cancer research: BCR. 2011;13(5):R86.
217. Aktas B, Muller V, Tewes M, Zeitz J, Kasimir-Bauer S, Loehberg CR, et al. Comparison of estrogen and progesterone receptor status of circulating tumor cells and the primary tumor in metastatic breast cancer patients. Gynecologic oncology. 2011;122(2):356-60.
218. Zurita M, Lara PC, del Moral R, Torres B, Linares-Fernandez JL, Arrabal SR, et al. Hypermethylated 14-3-3-sigma and ESR1 gene promoters in serum as candidate biomarkers for the diagnosis and treatment efficacy of breast cancer metastasis. BMC cancer. 2010;10:217.
219. Wong NS, Buckman RA, Clemons M, Verma S, Dent S, Trudeau ME, et al. Phase I/II trial of metronomic chemotherapy with daily dalteparin and cyclophosphamide, twice-weekly methotrexate, and daily prednisone as therapy for metastatic breast cancer using vascular endothelial growth factor and soluble vascular endothelial growth factor receptor levels as markers of response. Journal of clinical oncology: official journal of the American Society of Clinical Oncology. 2010;28(5):723-30.
220. Vo MN, Evans M, Leitzel K, Ali SM, Wilson M, Demers L, et al. Elevated plasma endoglin (CD105) predicts decreased response and survival in a metastatic breast cancer trial of hormone therapy. Breast cancer research and treatment. 2010;119(3):767-71.
221. Van der Auwera I, Peeters D, Benoy IH, Elst HJ, Van Laere SJ, Prove A, et al. Circulating tumour cell detection: a direct comparison between the CellSearch System, the AdnaTest and CK-19/mammaglobin RT-PCR in patients with metastatic breast cancer. British journal of cancer. 2010;102(2):276-84.
222. Theodoropoulos PA, Polioudaki H, Agelaki S, Kallergi G, Saridaki Z, Mavroudis D, et al. Circulating tumor cells with a putative stem cell phenotype in peripheral blood of patients with breast cancer. Cancer letters. 2010;288(1):99-106.
223. Tang Y, Wang L, Zhang P, Wei H, Gao R, Liu X, et al. Detection of circulating anti-mucin 1 (MUC1) antibodies in breast tumor patients by indirect enzyme-linked immunosorbent assay using a recombinant MUC1 protein containing six tandem repeats and expressed in Escherichia coli. Clinical and vaccine immunology: CVI. 2010;17(12):1903-8.
224. Shih NY, Lai HL, Chang GC, Lin HC, Wu YC, Liu JM, et al. Anti-α-enolase autoantibodies are down-regulated in advanced cancer patients. Japanese journal of clinical oncology. 2010;40(7):663-9.
225. Roth C, Rack B, Muller V, Janni W, Pantel K, Schwarzenbach H. Circulating microRNAs as blood-based markers for patients with primary and metastatic breast cancer. Breast cancer research: BCR. 2010;12(6):R90.
226. Punnoose EA, Atwal SK, Spoerke JM, Savage H, Pandita A, Yeh RF, et al. Molecular biomarker analyses using circulating tumor cells. PloS one. 2010;5(9):e12517.
227. Nakamura S, Yagata H, Ohno S, Yamaguchi H, Iwata H, Tsunoda N, et al. Multi-center study evaluating circulating tumor cells as a surrogate for response to treatment and overall survival in metastatic breast cancer. Breast cancer (Tokyo, Japan). 2010;17(3):199-204.
228. Munzone E, Nole F, Goldhirsch A, Botteri E, Esposito A, Zorzino L, et al. Changes of HER2 status in circulating tumor cells compared with the primary tumor during treatment for advanced breast cancer. Clinical breast cancer. 2010;10(5):392-7.
229. Lu J, Fan T, Zhao Q, Zeng W, Zaslavsky E, Chen JJ, et al. Isolation of circulating epithelial and tumor progenitor cells with an invasive phenotype from breast cancer patients. International journal of cancer. 2010;126(3):669-83.
230. Jensen AB, Wynne C, Ramirez G, He W, Song Y, Berd Y, et al. The cathepsin K inhibitor odanacatib suppresses bone resorption in women with breast cancer and established bone metastases: results of a 4-week, double-blind, randomized, controlled trial. Clinical breast cancer. 2010;10(6):452-8.
231. Hu Y, Fan L, Zheng J, Cui R, Liu W, He Y, et al. Detection of circulating tumor cells in breast cancer patients utilizing multiparameter flow cytometry and assessment of the prognosis of patients in different CTCs levels. Cytometry Part A: the journal of the International Society for Analytical Cytology. 2010;77(3):213-9.
232. Flores LM, Kindelberger DW, Ligon AH, Capelletti M, Fiorentino M, Loda M, et al. Improving the yield of circulating tumour cells facilitates molecular characterisation and recognition of discordant HER2 amplification in breast cancer. British journal of cancer. 2010;102(10):1495-502.
233. Fehm T, Muller V, Aktas B, Janni W, Schneeweiss A, Stickeler E, et al. HER2 status of circulating tumor cells in patients with metastatic breast cancer: a prospective, multicenter trial. Breast cancer research and treatment. 2010;124(2):403-12.
234. De Giorgi U, Valero V, Rohren E, Mego M, Doyle GV, Miller MC, et al. Circulating tumor cells and bone metastases as detected by FDG-PET/CT in patients with metastatic breast cancer. Annals of oncology: official journal of the European Society for Medical Oncology/ESMO. 2010;21(1):33-9.
235. De Giorgi U, Mego M, Rohren EM, Liu P, Handy BC, Reuben JM, et al. 18F-FDG PET/CT findings and circulating tumor cell counts in the monitoring of systemic therapies for bone metastases from breast cancer. Journal of nuclear medicine: official publication, Society of Nuclear Medicine. 2010;51(8):1213-8.
236. Daniele A, Zito AF, Giannelli G, Divella R, Asselti M, Mazzocca A, et al. Expression of metalloproteinases MMP-2 and MMP-9 in sentinel lymph node and serum of patients with metastatic and non-metastatic breast cancer. Anticancer research. 2010;30(9):3521-7.
237. Cheung KL, Iles RK, Robertson JF. Bony metastases from breast cancer - a study of foetal antigen 2 as a blood tumour marker. World journal of surgical oncology. 2010;8:38.
238. Botteri E, Sandri MT, Bagnardi V, Munzone E, Zorzino L, Rotmensz N, et al. Modeling the relationship between circulating tumour cells number and prognosis of metastatic breast cancer. Breast cancer research and treatment. 2010;122(1):211-7.
239. Board RE, Wardley AM, Dixon JM, Armstrong AC, Howell S, Renshaw L, et al. Detection of PIK3CA mutations in circulating free DNA in patients with breast cancer. Breast cancer research and treatment. 2010;120(2):461-7.
240. Bidard FC, Mathiot C, Degeorges A, Etienne-Grimaldi MC, Delva R, Pivot X, et al. Clinical value of circulating endothelial cells and circulating tumor cells in metastatic breast cancer patients treated first line with bevacizumab and chemotherapy. Annals of oncology: official journal of the European Society for Medical Oncology/ESMO. 2010;21(9):1765-71.
241. Voorzanger-Rousselot N, Journe F, Doriath V, Body JJ, Garnero P. Assessment of circulating Dickkopf-1 with a new two-site immunoassay in healthy subjects and women with breast cancer and bone metastases. Calcified tissue international. 2009;84(5):348-54.
242. Vazquez-Martin A, Fernandez-Real JM, Oliveras-Ferraros C, Navarrete JM, Martin-Castillo B, Del Barco S, et al. Fatty acid synthase activity regulates HER2 extracellular domain shedding into the circulation of HER2-positive metastatic breast cancer patients. International journal of oncology. 2009;35(6):1369-76.
243. Van der Auwera I, Elst HJ, Van Laere SJ, Maes H, Huget P, van Dam P, et al. The presence of circulating total DNA and methylated genes is associated with circulating tumour cells in blood from breast cancer patients. British journal of cancer. 2009;100(8):1277-86.
244. Tumminello FM, Badalamenti G, Incorvaia L, Fulfaro F, D’Amico C, Leto G. Serum interleukin-6 in patients with metastatic bone disease: correlation with cystatin C. Medical oncology (Northwood, London, England). 2009;26(1):10-5.
245. Todorovic-Rakovic N, Neskovic-Konstantinovic Z, Nikolic-Vukosavljevic D. Stage-related plasma values of transforming growth factor-beta1 are steroid receptors dependent. Clinical and experimental medicine. 2009;9(4):313-7.
246. Tewes M, Aktas B, Welt A, Mueller S, Hauch S, Kimmig R, et al. Molecular profiling and predictive value of circulating tumor cells in patients with metastatic breast cancer: an option for monitoring response to breast cancer related therapies. Breast cancer research and treatment. 2009;115(3):581-90.
247. Serrano MJ, Sanchez-Rovira P, Delgado-Rodriguez M, Gaforio JJ. Detection of circulating tumor cells in the context of treatment: prognostic value in breast cancer. Cancer biology & therapy. 2009;8(8):671-5.
248. Schaub NP, Jones KJ, Nyalwidhe JO, Cazares LH, Karbassi ID, Semmes OJ, et al. Serum proteomic biomarker discovery reflective of stage and obesity in breast cancer patients. Journal of the American College of Surgeons. 2009;208(5):970-8; discussion 8-80.
249. Pestrin M, Bessi S, Galardi F, Truglia M, Biggeri A, Biagioni C, et al. Correlation of HER2 status between primary tumors and corresponding circulating tumor cells in advanced breast cancer patients. Breast cancer research and treatment. 2009;118(3):523-30.
250. Payne RE, Yague E, Slade MJ, Apostolopoulos C, Jiao LR, Ward B, et al. Measurements of EGFR expression on circulating tumor cells are reproducible over time in metastatic breast cancer patients. Pharmacogenomics. 2009;10(1):51-7.
251. Mego M, De Giorgi U, Hsu L, Ueno NT, Valero V, Jackson S, et al. Circulating tumor cells in metastatic inflammatory breast cancer. Annals of oncology: official journal of the European Society for Medical Oncology/ESMO. 2009;20(11):1824-8.
252. Mego M, De Giorgi U, Broglio K, Dawood S, Valero V, Andreopoulou E, et al. Circulating tumour cells are associated with increased risk of venous thromboembolism in metastatic breast cancer patients. British journal of cancer. 2009;101(11):1813-6.
253. Martin M, Garcia-Saenz JA, Maestro De las Casas ML, Vidaurreta M, Puente J, Veganzones S, et al. Circulating tumor cells in metastatic breast cancer: timing of blood extraction for analysis. Anticancer research. 2009;29(10):4185-7.
254. Maestro LM, Sastre J, Rafael SB, Veganzones SB, Vidaurreta M, Martin M, et al. Circulating tumor cells in solid tumor in metastatic and localized stages. Anticancer research. 2009;29(11):4839-43.
255. Liu MC, Shields PG, Warren RD, Cohen P, Wilkinson M, Ottaviano YL, et al. Circulating tumor cells: a useful predictor of treatment efficacy in metastatic breast cancer. Journal of clinical oncology: official journal of the American Society of Clinical Oncology. 2009;27(31):5153-9.
256. Kim HS, Park YH, Park MJ, Chang MH, Jun HJ, Kim KH, et al. Clinical significance of a serum CA15-3 surge and the usefulness of CA15-3 kinetics in monitoring chemotherapy response in patients with metastatic breast cancer. Breast cancer research and treatment. 2009;118(1):89-97.
257. Kallergi G, Markomanolaki H, Giannoukaraki V, Papadaki MA, Strati A, Lianidou ES, et al. Hypoxia-inducible factor-1α and vascular endothelial growth factor expression in circulating tumor cells of breast cancer patients. Breast cancer research: BCR. 2009;11(6):R84.
258. Ibusuki M, Fujimori H, Yamamoto Y, Ota K, Ueda M, Shinriki S, et al. Midkine in plasma as a novel breast cancer marker. Cancer science. 2009;100(9):1735-9.
259. Hammad LA, Wu G, Saleh MM, Klouckova I, Dobrolecki LE, Hickey RJ, et al. Elevated levels of hydroxylated phosphocholine lipids in the blood serum of breast cancer patients. Rapid communications in mass spectrometry: RCM. 2009;23(6):863-76.
260. Goodale D, Phay C, Brown W, Gray-Statchuk L, Furlong P, Lock M, et al. Flow cytometric assessment of monocyte activation markers and circulating endothelial cells in patients with localized or metastatic breast cancer. Cytometry Part B, Clinical cytometry. 2009;76(2):107-17.
261. Finn RS, Gagnon R, Di Leo A, Press MF, Arbushites M, Koehler M. Prognostic and predictive value of HER2 extracellular domain in metastatic breast cancer treated with lapatinib and paclitaxel in a randomized phase III study. Journal of clinical oncology: official journal of the American Society of Clinical Oncology. 2009;27(33):5552-8.
262. De Giorgi U, Valero V, Rohren E, Dawood S, Ueno NT, Miller MC, et al. Circulating tumor cells and [18F]fluorodeoxyglucose positron emission tomography/computed tomography for outcome prediction in metastatic breast cancer. Journal of clinical oncology: official journal of the American Society of Clinical Oncology. 2009;27(20):3303-11.
263. Bramwell VH, Doig GS, Tuck AB, Wilson SM, Tonkin KS, Tomiak A, et al. Changes over time of extracellular domain of HER2 (ECD/HER2) serum levels have prognostic value in metastatic breast cancer. Breast cancer research and treatment. 2009;114(3):503-11.
264. Bianchi Ga, Loibl Sf, Zamagni Cd, Salvagni Se, Raab Gg, Siena Sc, et al. Phase II multicenter, uncontrolled trial of sorafenib in patients with metastatic breast cancer. Anti-Cancer Drugs. 2009;20(7):616-24.
265. Aktas B, Tewes M, Fehm T, Hauch S, Kimmig R, Kasimir-Bauer S. Stem cell and epithelial-mesenchymal transition markers are frequently overexpressed in circulating tumor cells of metastatic breast cancer patients. Breast cancer research: BCR. 2009;11(4):R46.
266. Yonemori K, Katsumata N, Noda A, Uno H, Yunokawa M, Nakano E, et al. Development and verification of a prediction model using serum tumor markers to predict the response to chemotherapy of patients with metastatic or recurrent breast cancer. Journal of cancer research and clinical oncology. 2008;134(11):1199-206.
267. Yildirim Y, Gunel N, Coskun U, Sancak B, Bukan N, Aslan S, et al. Serum big endothelin-1 levels in female patients with breast cancer. International immunopharmacology. 2008;8(8):1119-23.
268. Yildirim Y, Gunel N, Coskun U, Pasaoglu H, Aslan S, Cetin A. Serum neopterin levels in patients with breast cancer. Medical oncology (Northwood, London, England). 2008;25(4):403-7.
269. Yagata H, Nakamura S, Toi M, Bando H, Ohno S, Kataoka A. Evaluation of circulating tumor cells in patients with breast cancer: multi-institutional clinical trial in Japan. International journal of clinical oncology. 2008;13(3):252-6.
270. Velaiutham S, Taib NA, Ng KL, Yoong BK, Yip CH. Does the pre-operative value of serum CA15-3 correlate with survival in breast cancer? Asian Pacific journal of cancer prevention: APJCP. 2008;9(3):445-8.
271. Schrohl AS, Mueller V, Christensen IJ, Pantel K, Thomssen C, Bruenner N. A comparative study of tissue inhibitor of metalloproteinases-1 levels in plasma and tumour tissue from patients with primary breast cancer and in plasma from patients with metastatic breast cancer. Tumour biology: the journal of the International Society for Oncodevelopmental Biology and Medicine. 2008;29(3):181-7.
272. Saad A, Kanate A, Sehbai A, Marano G, Hobbs G, Abraham J. Correlation among [18F]fluorodeoxyglucose positron emission tomography/computed tomography, cancer antigen 27.29, and circulating tumor cell testing in metastatic breast cancer. Clinical breast cancer. 2008;8(4):357-61.
273. Plumer A, Duan H, Subramaniam S, Lucas FL, Miesfeldt S, Ng AK, et al. Development of fragment-specific osteopontin antibodies and ELISA for quantification in human metastatic breast cancer. BMC cancer. 2008;8:38.
274. Nole F, Munzone E, Zorzino L, Minchella I, Salvatici M, Botteri E, et al. Variation of circulating tumor cell levels during treatment of metastatic breast cancer: prognostic and therapeutic implications. Annals of oncology: official journal of the European Society for Medical Oncology/ESMO. 2008;19(5):891-7.
275. Molina R, Gion M, Gressner A, Troalen F, Auge JM, Holdenrieder S, et al. Alternative antibody for the detection of CA15-3 antigen: a European multicenter study for the evaluation of the analytical and clinical performance of the Access BR Monitor assay on the UniCel Dxl 800 Immunoassay System. Clinical chemistry and laboratory medicine. 2008;46(5):612-22.
276. Lipton A, Leitzel K, Chaudri-Ross HA, Evans DB, Ali SM, Demers L, et al. Serum TIMP-1 and response to the aromatase inhibitor letrozole versus tamoxifen in metastatic breast cancer. Journal of clinical oncology: official journal of the American Society of Clinical Oncology. 2008;26(16):2653-8.
277. Kyselova Z, Mechref Y, Kang P, Goetz JA, Dobrolecki LE, Sledge GW, et al. Breast cancer diagnosis and prognosis through quantitative measurements of serum glycan profiles. Clinical chemistry. 2008;54(7):1166-75.
278. Kallergi G, Agelaki S, Kalykaki A, Stournaras C, Mavroudis D, Georgoulias V. Phosphorylated EGFR and PI3K/Akt signaling kinases are expressed in circulating tumor cells of breast cancer patients. Breast cancer research: BCR. 2008;10(5):R80.
279. Generali D, Dovio A, Tampellini M, Tucci M, Tedoldi S, Torta M, et al. Changes of bone turnover markers and serum PTH after night or morning administration of zoledronic acid in breast cancer patients with bone metastases. British journal of cancer. 2008;98(11):1753-8.
280. Deng G, Herrler M, Burgess D, Manna E, Krag D, Burke JF. Enrichment with anti-cytokeratin alone or combined with anti-EpCAM antibodies significantly increases the sensitivity for circulating tumor cell detection in metastatic breast cancer patients. Breast cancer research: BCR. 2008;10(4):R69.
281. Dawood S, Broglio K, Valero V, Reuben J, Handy B, Islam R, et al. Circulating tumor cells in metastatic breast cancer: from prognostic stratification to modification of the staging system? Cancer. 2008;113(9):2422-30.
282. Cameron D, Casey M, Press M, Lindquist D, Pienkowski T, Romieu CG, et al. A phase III randomized comparison of lapatinib plus capecitabine versus capecitabine alone in women with advanced breast cancer that has progressed on trastuzumab: Updated efficacy and biomarker analyses. Breast Cancer Research & Treatment. 2008;112(3):533-43.
283. Burstein HJ, Chen YH, Parker LM, Savoie J, Younger J, Kuter I, et al. VEGF as a marker for outcome among advanced breast cancer patients receiving anti-VEGF therapy with bevacizumab and vinorelbine chemotherapy. Clinical cancer research: an official journal of the American Association for Cancer Research. 2008;14(23):7871-7.
284. Bidard FC, Vincent-Salomon A, Sigal-Zafrani B, Dieras V, Mathiot C, Mignot L, et al. Prognosis of women with stage IV breast cancer depends on detection of circulating tumor cells rather than disseminated tumor cells. Annals of oncology: official journal of the European Society for Medical Oncology/ESMO. 2008;19(3):496-500.
285. Ali SM, Carney WP, Esteva FJ, Fornier M, Harris L, Kostler WJ, et al. Serum HER-2/neu and relative resistance to trastuzumab-based therapy in patients with metastatic breast cancer. Cancer. 2008;113(6):1294-301.
286. Zhong XY, Ladewig A, Schmid S, Wight E, Hahn S, Holzgreve W. Elevated level of cell-free plasma DNA is associated with breast cancer. Archives of gynecology and obstetrics. 2007;276(4):327-31.
287. Tesarova P, Kalousova M, Trnkova B, Soukupova J, Argalasova S, Mestek O, et al. Carbonyl and oxidative stress in patients with breast cancer—is there a relation to the stage of the disease? Neoplasma. 2007;54(3):219-24.
288. Svane IM, Pedersen AE, Johansen JS, Johnsen HE, Nielsen D, Kamby C, et al. Vaccination with p53 peptide-pulsed dendritic cells is associated with disease stabilization in patients with p53 expressing advanced breast cancer; monitoring of serum YKL-40 and IL-6 as response biomarkers. Cancer immunology, immunotherapy: CII. 2007;56(9):1485-99.
289. Sandri MT, Johansson HA, Zorzino L, Salvatici M, Passerini R, Maisonneuve P, et al. Serum EGFR and serum HER-2/neu are useful predictive and prognostic markers in metastatic breast cancer patients treated with metronomic chemotherapy. Cancer. 2007;110(3):509-17.
290. Salem AM, Zohny SF, Abd El-Wahab MM, Hamdy R. Predictive value of osteocalcin and beta-CrossLaps in metastatic breast cancer. Clinical biochemistry. 2007;40(16-17):1201-8.
291. Riethdorf S, Fritsche H, Muller V, Rau T, Schindlbeck C, Rack B, et al. Detection of circulating tumor cells in peripheral blood of patients with metastatic breast cancer: a validation study of the CellSearch system. Clinical cancer research: an official journal of the American Association for Cancer Research. 2007;13(3):920-8.
292. Pollmann D, Siepmann S, Geppert R, Wernecke KD, Possinger K, Luftner D. The amino-terminal propeptide (PINP) of type I collagen is a clinically valid indicator of bone turnover and extent of metastatic spread in osseous metastatic breast cancer. Anticancer research. 2007;27(4a):1853-62.
293. Oremek G, Sauer-Eppel H, Klepzig M. Total procollagen type 1 amino-terminal propeptide (total P1NP) as a bone metastasis marker in gynecological carcinomas. Anticancer research. 2007;27(4a):1961-2.
294. Mountzios G, Dimopoulos MA, Bamias A, Papadopoulos G, Kastritis E, Syrigos K, et al. Abnormal bone remodeling process is due to an imbalance in the receptor activator of nuclear factor-κB ligand (RANKL)/osteoprotegerin (OPG) axis in patients with solid tumors metastatic to the skeleton. Acta oncologica (Stockholm, Sweden). 2007;46(2):221-9.
295. Lipton A, Ali SM, Leitzel K, Demers L, Evans DB, Hamer P, et al. Elevated plasma tissue inhibitor of metalloproteinase-1 level predicts decreased response and survival in metastatic breast cancer. Cancer. 2007;109(10):1933-9.
296. Konukoglu D, Turhan MS, Celik V, Turna H. Relation of serum vascular endothelial growth factor as an angiogenesis biomarker with nitric oxide & urokinase-type plasminogen activator in breast cancer patients. The Indian journal of medical research. 2007;125(6):747-51.
297. Gatt A, Makris A, Cladd H, Burcombe RJ, Smith JM, Cooper P, et al. Hyperhomocysteinemia in women with advanced breast cancer. International Journal of Laboratory Hematology. 2007;29(6):421-5.
298. Fehm T, Becker S, Duerr-Stoerzer S, Sotlar K, Mueller V, Wallwiener D, et al. Determination of HER2 status using both serum HER2 levels and circulating tumor cells in patients with recurrent breast cancer whose primary tumor was HER2 negative or of unknown HER2 status. Breast cancer research: BCR. 2007;9(5):R74.
299. Fabre-Lafay S, Monville F, Garrido-Urbani S, Berruyer-Pouyet C, Ginestier C, Reymond N, et al. Nectin-4 is a new histological and serological tumor associated marker for breast cancer. BMC cancer. 2007;7:73.
300. Eichbaum MH, de Rossi TM, Kaul S, Bruckner T, Schneeweiss A, Sohn C. Serum levels of hepatocyte growth factor/scatter factor in patients with liver metastases from breast cancer. Tumour biology: The journal of the International Society for Oncodevelopmental Biology and Medicine. 2007;28(1):36-44.
301. Cristofanilli M, Broglio KR, Guarneri V, Jackson S, Fritsche HA, Islam R, et al. Circulating tumor cells in metastatic breast cancer: biologic staging beyond tumor burden. Clinical breast cancer. 2007;7(6):471-9.
302. Colomer RMDP, Llombart-Cussac AMD, Lloveras BMD, Ramos MMD, Mayordomo JIMD, Fernandez RMD, et al. High circulating HER2 extracellular domain levels correlate with reduced efficacy of an aromatase inhibitor in hormone receptor-positive metastatic breast cancer: A confirmatory prospective study. Cancer. 2007;110(10):2178-85.
303. Byrne GJ, Hayden KE, McDowell G, Lang H, Kirwan CC, Tetlow L, et al. Angiogenic characteristics of circulating and tumoural thrombospondin-1 in breast cancer. International journal of oncology. 2007;31(5):1127-32.
304. Baskic D, Ristic P, Matic S, Bankovic D, Popovic S, Arsenijevic N. Clinical evaluation of the simultaneous determination of CA 15-3, CA 125 and sHER2 in breast cancer. Biomarkers: biochemical indicators of exposure, response, and susceptibility to chemicals. 2007;12(6):657-67.
305. Asgeirsson KS, Agrawal A, Allen C, Hitch A, Ellis IO, Chapman C, et al. Serum epidermal growth factor receptor and HER2 expression in primary and metastatic breast cancer patients. Breast cancer research: BCR. 2007;9(6):R75.
306. Al-Mowallad A, Kirwan C, Byrne G, McDowell G, Li C, Stewart A, et al. Vascular endothelial growth factor-C in patients with breast cancer. In vivo (Athens, Greece). 2007;21(3):549-51.
307. Zucker S, Wang M, Sparano JA, Gradishar WJ, Ingle JN, Davidson NE. Plasma matrix metalloproteinases 7 and 9 in patients with metastatic breast cancer treated with marimastat or placebo: Eastern Cooperative Oncology Group trial E2196. Clinical breast cancer. 2006;6(6):525-9.
308. Wong NS, Kahn HJ, Zhang L, Oldfield S, Yang LY, Marks A, et al. Prognostic significance of circulating tumour cells enumerated after filtration enrichment in early and metastatic breast cancer patients. Breast cancer research and treatment. 2006;99(1):63-9.
309. Witzel I, Thomssen C, Krenkel S, Wilczak W, Bubenheim M, Pantel K, et al. Clinical utility of determination of HER-2/neu and EGFR fragments in serum of patients with metastatic breast cancer. The International journal of biological markers. 2006;21(3):131-40.
310. Tampellini M, Berruti A, Bitossi R, Gorzegno G, Alabiso I, Bottini A, et al. Prognostic significance of changes in CA 15-3 serum levels during chemotherapy in metastatic breast cancer patients. Breast cancer research and treatment. 2006;98(3):241-8.
311. Silva HC, Garcao F, Coutinho EC, De Oliveira CF, Regateiro FJ. Soluble VCAM-1 and E-selectin in breast cancer. Neoplasma. 2006;53(6):538-43.
312. Pectasides D, Gaglia A, Arapantoni-Dadioti P, Bobota A, Valavanis C, Kostopoulou V, et al. HER-2/neu status of primary breast cancer and corresponding metastatic sites in patients with advanced breast cancer treated with trastuzumab-based therapy. Anticancer research. 2006;26(1b):647-53.
313. Palmieri C, MacGregor T, Girgis S, Vigushin D. Serum 25-hydroxyvitamin D levels in early and advanced breast cancer. Journal of clinical pathology. 2006;59(12):1334-6.
314. Ntoulia M, Stathopoulou A, Ignatiadis M, Malamos N, Mavroudis D, Georgoulias V, et al. Detection of Mammaglobin A-mRNA-positive circulating tumor cells in peripheral blood of patients with operable breast cancer with nested RT-PCR. Clinical biochemistry. 2006;39(9):879-87.
315. Meng S, Tripathy D, Shete S, Ashfaq R, Saboorian H, Haley B, et al. uPAR and HER-2 gene status in individual breast cancer cells from blood and tissues. Proceedings of the National Academy of Sciences of the United States of America. 2006;103(46):17361-5.
316. Leto G, Incorvaia L, Badalamenti G, Tumminello FM, Gebbia N, Flandina C, et al. Activin A circulating levels in patients with bone metastasis from breast or prostate cancer. Clinical & experimental metastasis. 2006;23(2):117-22.
317. Hayes DF, Cristofanilli M, Budd GT, Ellis MJ, Stopeck A, Miller MC, et al. Circulating tumor cells at each follow-up time point during therapy of metastatic breast cancer patients predict progression-free and overall survival. Clinical cancer research: an official journal of the American Association for Cancer Research. 2006;12(14 Pt 1):4218-24.
318. Granato AM, Frassineti GL, Giovannini N, Ballardini M, Nanni O, Maltoni R, et al. Do serum angiogenic growth factors provide additional information to that of conventional markers in monitoring the course of metastatic breast cancer? Tumour biology: the journal of the International Society for Oncodevelopmental Biology and Medicine. 2006;27(6):302-8.
319. Budd GT, Cristofanilli M, Ellis MJ, Stopeck A, Borden E, Miller MC, et al. Circulating tumor cells versus imaging—predicting overall survival in metastatic breast cancer. Clinical cancer research: an official journal of the American Association for Cancer Research. 2006;12(21):6403-9.
320. Benoy IH, Elst H, Philips M, Wuyts H, Van Dam P, Scharpe S, et al. Real-time RT-PCR detection of disseminated tumour cells in bone marrow has superior prognostic significance in comparison with circulating tumour cells in patients with breast cancer. British journal of cancer. 2006;94(5):672-80.

## Appendix D. Detailed Overview of All Biomarkers per Developmental Stage

For all biomarkers an overview was created of the number of studies that investigated the biomarker and the translational stages in which these studies have been performed. Some studies focused on biomarkers from multiple categories (e.g., they focused on cells and proteins at the same time). All biomarkers investigated in these studies were categorized according to their main research subject. If the article for example mainly studied CTCs but also measured the concentration of CA15-3, then this study was classified in the cells category. In the following sections an overview of all biomarkers that were classified in the four general biomarker categories is presented.

### Appendix D.1. Cells

Several types of research on cells as blood-based biomarkers have been performed. Studies enumerated cells, investigated the expression of proteins on their membrane or investigated the expression of genes that extracted from DNA or mRNA of the cell. The first type of studies are those investigating the enumeration of whole cells or cell clusters. All cells or cell clusters that have been investigated are presented in Table A1.

The second type of studies that investigated cells are those studies that investigate the proteins that are expressed on the membranes of the cells. Table A2 presents the proteins that have been investigated on the membranes of cells. The fourth column “Gene” presents the abbreviations of the genes that encode for these proteins. The fifth column “Gene ID” presents the gene ID of the gene that encodes for the membrane protein.

The third type of studies included in the cells category presented research on the gene expression within these cells. Table A3 presents the genes have been investigated, after DNA or mRNA was extracted from cells.

Studies that mainly focus on cells have also investigated a couple of proteins parallel to their research on the enumeration of cells, membrane expression of proteins on those cells or gene expression within those cells. Table A4 presents the proteins that have been investigated in studies that mainly focused on cells.

Besides the above presented blood-based biomarkers, in one study microRNA 10b had been investigated in parallel with research on CTCs (basic predictive research). In five other studies DNA from cells was extracted and whole genome amplification was performed (all 5 studies were categorized as basic predictive research).

### Appendix D.2. Proteins

Several types of proteins have been investigated. Table A5 presents an overview of all proteins that have been investigated in studies which mainly focus on proteins as blood-based biomarkers.

In these studies the main research aim is on proteins as blood-based biomarkers. However, also a couple of other biomarkers have been investigated. In two studies they simultaneously investigated the enumeration of CTCs. In eight studies they looked at gene expression patterns within DNA or mRNA. The genes investigated in these studies were *OPG*, *RANKL*, *eNOS*, and *THBS-1*.

### Appendix D.3. DNA

Table A6 presents al genes that have been investigated in studies that mainly focus on circulating DNA.

Besides DNA investigated, several articles investigated proteins or cells in parallel. Two studies enumerated CTCs in parallel (basic prognostic research). In 13 studies proteins were investigated in parallel. Seven studies focused on CA15-3, five on CEA and 1 on the plasminogen activator.

### Appendix D.4. RNA

Table A7 presents al circulating micro RNAs that have been studied.

Studies that mainly focused on microRNAs investigated the enumeration of CTCs in parallel in two studies, and the concentration of secreted Mammaglobin in one study.

**Table A1:** Translational stages of research on the enumeration of whole cells or cell clusters.

Biomarker Abbreviation	Biomarker	Total Number of Articles	Basic Research	Proof of Concept	Comparison of Methods	Basic Predictive Research	Basic Prognostic Research	Prognostic Validation	Observational	Trials	Health Economic Analyses
CAMLs	Cancer associated macrophage like cells	1				1					
CAFs	Cancer associated fibroblasts	1		1							
Monocyte CD63	Type of white blood cell CD63	1				1					
Monocyte CD64	Type of white blood cell CD64	1				1					
CECs	Circulating epithelial cells	2							2		
CEPs	Circulating epithelial progenitor cells	2							2		
CETC	Circulating epithelial tumor cells	2				1			1		
aCTCs	Apoptotic circulating tumor cells	1					1				
CSCs	Cancer Stem Cells	2				1			1		
CTC	Circulating tumor cells	156	4	19	20	46	35	1	29	2	
CTC Cluster	Circulating tumor cell clusters	3		1		1			1		
NKs	Natural Killer Cells	2				2					

**Table A2:** Translational stages of research on the membrane expression of proteins.

Biomarker Abbreviation	Biomarker	Cell *	Gene	Gene ID	Total Number of Articles	Basic Research	Proof of Concept	Comparison of Methods	Basic Predictive Research	Basic Prognostic Research	Prognostic Validation	Observational	Trials	Health Economic Analyses
Akt2	AKT serine theronine kinase 2	CTC	*AKT2*	208	4			1	1	1		1		
CD133	Prominin 1	CTC	*PROM1*	8842	1			1						
CD44	CD44 molecule	CTC	*CD44*	960	1		1							
ER	Estrogen	CTC	*ESR1*	2099	13	1	2	1	6	3				
Fibronectin	Fibronectin	CTC	*FN1*	2335	1				1					
HER 2	Human epidermal growth factor receptor 2	CTC	*ERBB2*	2064	25	1	1	3	8	4	0	8		
*N*-Cadherin	Cadherin 2	CTC	*CDH2*	1000	3			1	2					
PR	Progesteron	CTC	*PGR*	5241	7	1			4	2				
VEGF	Vascular endothelial growth factor	CTC	*VEGFA*	7422	4				2			2		
VEGFR2	Vascular endothelial growth factor receptor 2	CTC	*KDR*	3791	1							1		
Vimentin	Vimentin	CTC	*VIM*	7431	6			1	4	1				
CD24	CD24 molecule	Granulocytes	*CD24*	100133941	1		1							
TLR2	Toll Like Receptor 2	Lymphocytes	*TLR2*	7097	1				1					
TLR4	Toll Like Receptor 4	Lymphocytes	*TLR4*	7099	1				1					

* Cells given in this column represent the cells in which these biomarkers were found.

**Table A3:** Translational stages of research on gene expression within cell DNA or mRNA.

Biomarker Abbreviation	Biomarker	Cell *	Gene	Gene ID	Total Number of Articles	Basic Research	Proof of Concept	Comparison of Methods	Basic Predictive Research	Basic Prognostic Research	Prognostic Validation	Observational	Trials	Health Economic Analyses
CD34	CD34 Molecule	CSCs	*CD34*	947	1				1					
Nanog	NANOG	CSCs	*NANOG*	79923	1				1					
Nestin	Nestin	CSCs	*NES*	10763	1				1					
Oct3/4	POU class 5 homeobox 1	CSCs	*POU5F1*	5460	1				1					
Sox2	SRY Box 2	CSCs	*SOX2*	6657	1				1					
ACTA1	Actin/α 1, skeletal muscle	CTC	*ACTA1*	58	1				1					
AGR2	Anterior gradient 2 protein disulphide isomerase family member	CTC	*AGR2*	10551	1				1					
ALDH1	Aldehyde dehydrogenase 1 family member A1	CTC	*ALDH1A1*	216	5		1	1		2		1		
AURKA	Aurora Kinase A	CTC	*AURKA*	6790	1					1				
BCL2	BCL2 apoptosis regulator	CTC	*BCL2*	596	1		1							
BIRC5	baculoviral IAP repeat containing 5	CTC	*BIRC5*	332	1				1					
CCND1	Cyclin D1	CTC	*CCND1*	595	1				1					
CDC6	Cell Division Cycle 6	CTC	*CDC6*	990	1		1							
CENPF	Centromere protein F	CTC	*CENPF*	1063	1		1							
CEP55	Centrosomal protein 55	CTC	*CEP55*	55165	2		1		1					
CK19	Keratin type I cytoskeletal 19	CTC	*KRT19*	3880	6				4	1		1		
CK8	Cytokeratin 8	CTC	*KRT8*	3856	1				1					
CRABP2	Cellular retinoic acid binding protein 2	CTC	*CRABP2*	1382	1				1					
CSt6 promotor	Cystatin E/M	CTC	*CST6*	1474	1				1					
CXCL14	CXC motif chemokine ligand 14	CTC	*CXCL14*	9547	2				2					
CXXC5	CXXC finger protein 5	CTC	*CXXC5*	51523	1		1							
DTX3	Deltex E3 ubiquitin ligase 3	CTC	*DTX3*	196403	1				1					
DUSP4	Dual specificity phosphatase 4	CTC	*DUSP4*	1846	1				1					
EEF1A2	Eukaryotic translation elongation factor 1 α 2	CTC	*EEF1A2*	1917	2				2					
EGFR	Epidermal growth factor receptor	CTC	*EGFR*	1956	6		1		2	1		2		
ERBB3	ERB-b2 receptor tyrosine kinase 3	CTC	*ERBB3*	2065	2				1	1				
ERBB4	ERB-b2 receptor tyrosine kinase 4	CTC	*ERBB4*	2066	1				1					
ERCC1	ERCC excision repair 1, endonuclease non-catalytic subunit	CTC	*ERCC1*	2067	1					1				
ESR1	Estrogen Receptor 1	CTC	*ESR1*	2099	3		1		2					
FGFR4	Fibroblast gorwth factor receptor 4	CTC	*FGFR4*	2264	2		1		1					
FKBP10	FK506 binding protein 10	CTC	*FKBP10*	60681	1				1					
FOX A1	Forkhead box A1	CTC	*FOX A1*	3169	1				1					
FOXC1	Forkhead box C1	CTC	*FOXC1*	2296	1		1							
GAPDH	Glyceraldehyde-3-phosphate dehydrogenase	CTC	*GAPDH*	2507	1							1		
HER2	Human epidermal growth factor receptor 2	CTC	*ERBB2*	2064	17	1	3	2	9	1		1		
HIF-1a	hypoxia inducible factor-1 α	CTC	*HIF1A*	3091	1				1					
IGFBP2	Insulin like growth factor binding protein 2	CTC	*IGFBP2*	3485	1				1					
IGFBP4	Insulin like growth factor binding protein 4	CTC	*IGFBP4*	3487	1				1					
IL17 BR	Interleukin 17 receptor B	CTC	*IL17RB*	55540	1				1					
ITGA6	Integrin subunit α	CTC	*ITGA6*	3655	1				1					
Ki67	(proliferation marker)	CTC	*MKI67*	4288	1				1					
KRT14	Keratin 14	CTC	*KRT14*	3861	1		1							
KRT17	Keratin 17	CTC	*KRT17*	3872	1				1					
KRT19	Keratin 19	CTC	*KRT19*	3880	2				2					
KRT20	Keratin type I cytoskeletal 20	CTC	*KRT20*	54474	1		1							
KRT7	Keratin 7	CTC	*KRT7*	3855	1				1					
KRT81	Keratin 81	CTC	*KRT81*	3887	1				1					
LAD1	Ladinin 1	CTC	*LAD1*	3898	1				1					
Mamma-globin	Secretoglobin family 2a member 2	CTC	*SCGB2A2*	4250	3		1		2					
MELK	Maternal embryonic leucine zipper kinase	CTC	*MELK*	9833	1				1					
MUC1	CA15-3	CTC	*MUC1*	4582	5	1	1		2			1		
MYBL2	MYB proto-oncogene like 2	CTC	*MYBL2*	4605	1		1							
NDC80	NDC80 Kinetochore complex component	CTC	*NDC80*	10403	1		1							
NUF2	NUF2, NDC80 kinetochore complex component	CTC	*NUF2*	83540	1		1							
PIK3CA	Phosphalidylinositol-4,5-bisphosphate 3 kinase catalytic subunit α	CTC	*PIK3CA*	5290	7		1		4	1		1		
PIP	Prolactin induced protein	CTC	*PIP*	5304	1				1					
PKP3	Plakophilin 3	CTC	*PKP3*	11187	1				1					
PTPRK	Protein tyrosine phosphatase, receptor type K	CTC	*PTPRK*	5796	1				1					
PTRF	Polymerase I and transcript release factor	CTC	*PTRF*	284119	2				2					
PTTG1	Pituitary tumor transforming 1	CTC	*PTTG1*	9232	1		1							
RRM2	Ribonucleotide reductase regulatory subunit M2	CTC	*RRM2*	6241	1		1							
S100A7	S100 calcium binding protein A7	CTC	*S100A7*	6278	1				1					
SCGB1D2	Secretoglobin family 1D member 2	CTC	*SCGB1D2*	10647	1				1					
SLUG	Snail family transcriptional repressor 2	CTC	*SNAI2*	6591	1							1		
SNAIL1	Snail family transcriptional repressor 1	CTC	*SNAI1*	6615	1							1		
SPDEF	SAM Pointed domain containing ETS transcription factor	CTC	*SPDEF*	25803	1				1					
TFF3	Trefoil factor 3	CTC	*TFF3*	7033	2		1		1					
TMEM45B	Transmembrane protein 45B	CTC	*TMEM45B*	120224	1		1							
TSPAN13	Tetraspanin 13	CTC	*TSPAN13*	27075	1				1					
TWIST	TWIST	CTC	*TWIST1*	7291	7			3	2			2		
TYMS	Thymidylate synthesase	CTC	*TYMS*	7298	1		1							
UBE2C	Ubiquitin conjugating enzyme E2 C	CTC	*UBE2C*	11065	1		1							
UBE2T	Ubiquitin conjugating enzyme E2 T	CTC	*UBE2T*	29089	1		1							
uPAR	Tyrokinase plasminogen activator receptor	CTC	*PLAU*	5328	2				2					
TP53	Tumor Protein P53	CTC	*TP53*	7157	1				1					
MRP1	ATP binding cassette subfamily C member 1	CTC	*ABCC1*	4363	1				1					
MRP2	ATP binding cassette subfamily C member 2	CTC	*ABCC2*	1244	1				1					
CK18	Cytokeratin 18	CTC	*KRT18*	3875	1				1					
TFF1	Trefoil factor 1	CTC	*TFF1*	7031	2		1		1					
BMS1	BMS1 ribosome biogenesis factor	CTC	*BMS1*	9790	1				1					
SOX 17	SRY Box 17	CTC	*SOX17*	64321	1				1					

* Cells given in this column represent the cells in which these biomarkers were found.

**Table A4:** Translational stages of research on proteins investigated in parallel in studies which mainly focus on cells.

Biomarker Abbreviation	Biomarker	Membrane or Secreted Protein	Gene	Gene ID	Total Number of Articles	Basic Research	Proof of Concept	Comparison of Methods	Basic Predictive Research	Basic Prognostic Research	Prognostic Validation	Observational	Trials	Health Economic Analyses
HER2	Human epidermal growth factor receptor 2	Membrane	*MUC1*	4582	3				2	1				
pFAK	phosphorylated-focal adhesion kinase	Membrane	*PTK2*	5747	1				1					
CA15-3	CA15-3 , produced by MUC1	Secreted	*MUC1*	4582	17	1		2	4	4	1	5		
CAIX	Carbonic anhydrase IX	Secreted	*CA9*	768	1					1				
CEA	Carcinoembryonic antigen related cell adhesion molecule	Secreted	*CEACAM5*	1048	6	1		1	1	1	1	1		
CXCL1	chemokine (C-X-C Motif) Ligand-1	Secreted	*CXCL1*	2919	1					1				
Fibrinogen	Fibrinogen	Secreted	*FGA*	2243	1				1					
LDHA	Lactate dehydrogenase	Secreted	*LDHA*	3939	2				1			1		
M30	Cytokeratin 18 fragments	Secreted	*KRT18*	3875	3				1			2		
P53	Tumor Protein P53	Secreted	*TP53*	7157	1							1		
TGF-B	Transcription Growth Factor Beta 1	Secreted	*TGFB1*	7040	1					1				
TIMP-1	TIMP metallopeptidase inhibitor 1	Secreted	*TIMP1*	7076	1					1				
Bcl-2	BCL2 apoptosis regulator	Secreted	*BCL2*	596	1							1		

**Table A5:** Translational stages of proteins.

Biomarker Abbreviation	Biomarker	Type of Biomarker	Gene	Gene ID	Total Number of Articles	Basic Research	Proof of Concept	Comparison of Methods	Basic Predictive Research	Basic Prognostic Research	Prognostic Validation	Observational	Trials	Health Economic Analyses
EGFR	Epidermal growth factor receptor	Membrane	*EGFR*	1956	9			1	2	2		4		
ENDO180	Mannose Receptor, C type 2 (Endo 180)	Membrane	*MRC2*	9902	1				1					
Endoglin	Glycoprotein co-receptor for peptides in the TGF family	Membrane	*ENG*	2022	1							1		
E-selectin	Selectin E (SELE)	Membrane	*SELE*	6401	2				1			1		
HER2	Soluble Human epidermal growth factor receptor 2	Membrane	*ERBB2*	2064	19				5	8		6		
Jagged 1	Jagged 1	Membrane	*NOTCH1*	4851	1					1				
PDGFR-α	Platelet derived growth factor receptor α	Membrane	*PDGFRA*	5156	1							1		
RAGE	soluble receptor for advanced glycation end products (sRAGE)	Membrane	*AGER*	177	1				1					
RCF	Red Cell Folate	Membrane	*FOLR1*	2348	1				1					
sKIT	Stem cell factor receptor/KIT proto-oncogene receptor tyrosine kinase	Membrane	*KIT*	3815	1							1		
VEGFR-1	Vascular Endothelial Growth Factor Receptor 1	Membrane	*FLT1*	2321	1				1					
VEGFR-1	Vascular Endothelial Growth Factor Receptor 1	Membrane	*FLT1*	2321	2							2		
VEGFR-1	Vascular Endothelial Growth Factor Receptor 1	Membrane	*FLT1*	2321	2							2		
VEGFR-2	Vascular Endothelial Growth Factor Receptor 2	Membrane	*KDR*	3791	1				1					
VEGFR-2	Vascular Endothelial Growth Factor Receptor 2	Membrane	*KDR*	3791	3							3		
VEGFR-2	Vascular Endothelial Growth Factor Receptor 2	Membrane	*KDR*	3791	4				1			3		
VEGFR-3	Vascular endothelial growth factor receptor 3	Membrane	*FLT4*	2324	1							1		
a2-HS-glyco-protein	fetuin-A	Secreted	*AHSG*	197	1				1					
Activin A	Inhibin beta A subunit	Secreted	*INHBA*	3624	1				1					
ALP	serum Alkaline Phosphatase	Secreted	*ALPL*	249	4			1	2			1		
bFGF	Fibroblast growth factor 2	Secreted	*FGF2*	2247	1				1					
big ET-1	big endothelin 1-growth factor	Secreted	*EDN1*	1906	1				1					
CA19-9	Carbohydrate antigen	Secreted			2			1	1					
CAIX	Carbonic anhydrase IX	Secreted	*CA9*	768	2					1		1		
CEA	Carcinoembryonic antigen (CEA)	Secreted	*CEACAM5*	1084	8			1	7					
CML	Carboxymethyllysine	Secreted	*BCR*	613	1				1					
CRP	C-reactive protein	Secreted	*CRP*	1401	1					1				
CTX	C-terminal telopeptide	secreted	*CYP27A1*	1593	3				1			2		
CYFRA21-1	Cytokeratin 19 Fragment	Secreted	*KRT19*	3880	1				1					
Cyst C	inhibitor of IL-6	Secreted			1				1					
DKK-1	Dickkopf-1 (DKK1)	Secreted	*DKK1*	22943	2			1	1					
ENO1 ABS	Antibodies against human α enolase	Secreted	*ENO1*	2023	1				1					
ES	Endostatin	Secreted	*COL18A1*	80781	1							1		
FASN	Fatty acid synthase	Secreted	*FASN*	2194	2		1		1					
FSIP1	Fibrous sheath interacting protein	Secreted	*FSIP1*	161835	2				2					
Galectin-1	Galectin-1	Secreted	*LGALS1*	3956	1				1					
Galectin-8	Galectin-8	Secreted	*LGALS8*	3964	1				1					
GASP-1	G-protein coupled receptor associated sorting protein 1	Secreted	*GPRASP1*	9737	1				1					
G-CSF	Granulyte colony stimulating factor	Secreted	*CSF3R*	1441	1				1					
GDF15	Growth factor differentiation factor 15	Secreted	*GDF15*	9518	1				1					
GGT	Gamma-Glutamyltransferase	Secreted	*GGT1*	2678	1					1				
HMGB1	soluble high mobility group box 1 (HMGB1)	Secreted	*HMGB1*	3146	1				1					
HSP70	Heat shock protein 70	Secreted	*HSPA4*	3308	1							1		
IFN-y	Interferon gamma	Secreted	*IFNG*	3458	1					1				
IL-10	Interleukin 10	Secreted	*IL10*	3586	1					1				
IL-18	Interleukin 18	Secreted	*IL18*	3606	1			1						
IL-2	Interleukin 2	Secreted	*IL2*	3558	1					1				
IL-4	Interleukin 4	Secreted	*IL4*	3505	1					1				
IL-6	Interleukin 6	Secreted	*IL6*	3569	4				2	2				
IL-7	Interleukin 7	Secreted	*IL7*	3574	1				1					
IL-8	CXC motif chemokine ligand 8	Secreted	*CXCL8*	3576	1							1		
LDHA	Lactate dehydrogenase	Secreted	*LDHA*	3939	1				1					
M30	Cytokeratin 18 fragments	Secreted	*KRT18*	3875	1				1					
Midkine	Growth factor	Secreted	*MDK*	4192	1			1						
MMP2	Matrix Metallopeptidase 2	Secreted	*MMP2*	4313	1				1					
MMP7	Matrix Metallopeptidase 7	Secreted	*MMP7*	4316	1							1		
MMP9	Matrix Metalloproteinase 9	Secreted	*MMP9*	4318	2				1			1		
MUC1	Cancer Antigen 15-3	Secreted	*MUC1*	4582	21		1	4	13	2		1		
MUC1	Antigen	Secreted	*MUC1*	4582	1			1						
MUC16	Mucin 16 or Cancer antigen 125 or	Secreted	*MUC16*	94025	3				3					
Nectin-4	Nectin Cell Adhesion molecule 4	Secreted	*NECTIN4*	81607	1				1					
NSE	Neuron specific enolase	Secreted	*ENO2*	2026	1				1					
OC	Osteocalcin (bone gamma-carboxylglutamate) protein	Secreted	*BGLAP*	632	3				2			1		
OPG	Osteoprotegerin	Secreted	*TNFRSF11B*	4982	1				1					
PAI-1	Plasminogen activator inhibitor 1	Secreted	*SERPINE1*	5054	1							1		
Pik3CA	phosphatidylinositol-4,5-bisphosphate 3-kinase catalytic subunit α	Secreted	*PIK3CA*	5290	1							1		
PLG	Plasminogen	Secreted	*PLG*	5340	1				1					
Prothrombin	Coagulation factor II	Secreted	*F2*	2147	1				1					
PTH	Parathyroid hormone	Secreted	*PTH*	5741	1							1		
RANKL	Receptor activator of nuclear factor kappa-B ligand	Secreted	*TNFSF11*	8600	1				1					
Survivin	baculoviral IAP repeat containing 5	Secreted	*BIRC5*	332	1				1					
TATI	Tumor associated trypsin inhibitor	Secreted	*SPINK*	6690	1			1						
TGF-B1	Transcription Growth Factor Beta 1	Secreted	*TGFB1*	7040	4				1	1		2		
THBS-1	Thrombospondin (TSP-1)	Secreted	*THBS1*	7057	5				2			3		
TIMP1	Tissue inhibitor of metalloproteinase 1	Secreted	*TIMP1*	7076	3				2			1		
TK1	Thymidine kinase1	Secreted	*TK1*	7083	2				1	1				
TNC	Tenascin-C	Secreted	*TNC*	3371	1				1					
TPA	Tissue polypeptide antigen Plasminogen activator, tissue type	Secreted	*PLAT*	5327	1				1					
TRACP5a	Tartrate resistant acid phosphatase 5a	Secreted	*ACP5*	54	2				1	1				
TSH	Thyroid stimulating hormone	Secreted	*TSHB*	7252	1							1		
TWEAK	Tumor necrosis factor related weak inducer of apoptosis	Secreted	*TNFSF12*	8742	1				1					
u-PAR	Urokinase-type plasminogen activator	Secreted	*PLAU*	5328	1				1					
VEGF	Vascular Endothelial Growth Factor	Secreted	*VEGFA*	7422	11				4			7		
VEGF-A	Vascular Endothelial Growth Factor A	Secreted	*VEGFA*	7422	5							5		
VEGF-C	Vascular Endothelial Growth Factor C	Secreted	*VEGFC*	7424	2				1			1		
YKL-40	Chitinase-3-like protein 1	Secreted	*CHI3L1*	1116	2				2					
1CTP	C-terminal telopeptide of collagen type I	Secreted	*CYP27A1*	1593	1				1					
Fibrin α	fibrin alfa	Secreted	*FGA*	2243	1				1					
OPN	Osteopontin (Secreted phosphoprotein 1)	Secreted	*SPP1*	6696	2			1	1					
Osteonectin	Osteonectin	Secreted	*SPARC*	6678	1				1					
Periostin	Periostin	Secreted	*POSTN*	10631	1				1					
Phosphocholine	lipoprotein	Secreted	*PCYT1A*	5130	1		1							
VCAM1	Vascular endothelial adhesion molecule	Secreted	*VCAM1*	7412	1				1					

**Table A6:** Translational stage of genes investigated in studies that focus on circulating DNA.

Biomarker Abbreviation	Biomarker	Gene	Gene ID	Total Number of Articles	Basic Research	Proof of Concept	Comparison of Methods	Basic Predictive Research	Basic Prognostic Research	Prognostic Validation	Observational	Trials	Health Economic Analyses
AKR1B1	Aldo-keto reductase family 1	*AKR1B1*	231	1				1					
AKT1	AKT serine threonine kinase 1	*AKT1*	207	1				1					
APC	APC, WNT signaling pathway	*APC*	324	1				1					
ARHGEF7	Rho guanine nucleotide exchange factor 7	*ARHGEF7*	8874	1				1					
COL6A2	Collagen type VI α 2 chain	*COL6A2*	1292	1				1					
ESR1	Estrogen Receptor 1	*ESR1*	2099	4				4					
FGFR1	Fibroblast growth factor receptor 1	*FGFR1*	2260	1				1					
FGFR2	Fibroblast growth factor receptor 2	*FGFR2*	2263	1				1					
GPX7	Glutathione peroxidase 7	*GPX7*	2882	1				1					
GSTP1	Glutathione S-transferase pi1	*GSTP1*	2950	1			1						
HER2	Human epidermal growth factor receptor 2	*ERBB2*	2064	2				2					
HIST1H3C	Histone cluster 1 H3 family member C	*HIST1H3C*	8352	1				1					
HOXB4	Homeobox B4	*HOXB4*	3214	1				1					
IDH2	Isocitrate dehydrogenase 2	*IDH2*	3418	1				1					
KU86	X-ray repair cross complementin 5	*XRCC5*	7520	1						1			
PIK3CA	Phosphalidylinositol-4,5-bisphosphate 3 kinase catalytic subunit α	*PIK3CA*	5290	6				6					
PTEN	Phosphatase and tensin homolog	*PTEN*	5728	1				1					
*RARβ2* gene	Retinoic acid receoptor beta	*RARB*	5915	1			1						
RASGRF2	Ras protein specific guanine nucleotide-releasing factor 2	*RASGRF2*	5924	1				1					
RASSF1A	Ras association domain family member 1	*RASSF1*	11186	6			1	5					
Stratifin	Stratifin	*SFN*	2810	1				1					
TM6SF1	Transmembrane 6 superfamily member 1	*TM6F1*	53346	1				1					
TMEFF2	Transmembrane protein with EGF like and two follistatin like domains 2	*TMEFF2*	23671	1				1					
TP53	Tumor Protein P53	*TP53*	7157	5				4	1				
WGA	Whole genome amplification	*	*	1				1					

* Whole genome amplification has been performed once. No specific gene or gene ID has been reported as multiple genes can be identified. However, in the study that presented whole genome amplification, no further gene information has been presented.

**Table A7:** Translational stages of microRNAs.

Biomarker	Total Number of Articles	Basic Research	Proof of Concept	Comparison of Methods	Basic Predictive Research	Basic Prognostic Research	Prognostic Validation	Observational	Trials	Health Economic Analyses
miR-10b	4				3	1				
miR-1260	1				1					
miR-1280	1				1					
miR-141	2				1	1				
miR-155	3				3					
miR-16	2				2					
miR-17	1				1					
miR-197	1				1					
miR-19a	1					1				
miR-200a	1					1				
miR-200b	1					1				
miR-200c	2					2				
miR-2015	1				1					
miR-203	1					1				
miR-21	1					1				
miR-21	1				1					
miR-29b2	1				1					
miR-34a	2				2					
miR-373	1				1					
miR-41	1					1				
miR-720	1				1					
miR-93	1				1					
Profiling 65 miRs	1			1						
U6/SNORD44	1				1					
